# Dadasnake, a Snakemake implementation of DADA2 to process amplicon sequencing data for microbial ecology

**DOI:** 10.1093/gigascience/giaa135

**Published:** 2020-11-30

**Authors:** Christina Weißbecker, Beatrix Schnabel, Anna Heintz-Buschart

**Affiliations:** Helmholtz Centre for Environmental Research GmbH - UFZ, Department of Soil Ecology; Theodor-Lieser-Str. 4, 06120 Halle, Germany; Helmholtz Centre for Environmental Research GmbH - UFZ, Department of Soil Ecology; Theodor-Lieser-Str. 4, 06120 Halle, Germany; Helmholtz Centre for Environmental Research GmbH - UFZ, Department of Soil Ecology; Theodor-Lieser-Str. 4, 06120 Halle, Germany; German Centre for Integrative Biodiversity Research (iDiv) Halle-Jena-Leipzig, Metagenomics Support Unit; Puschstr. 4, 04103 Leipzig, Germany

**Keywords:** rRNA gene sequence analysis, denoising, exact sequence variants, R, pipeline, microbiome, community structure

## Abstract

**Background:**

Amplicon sequencing of phylogenetic marker genes, e.g., 16S, 18S, or ITS ribosomal RNA sequences, is still the most commonly used method to determine the composition of microbial communities. Microbial ecologists often have expert knowledge on their biological question and data analysis in general, and most research institutes have computational infrastructures to use the bioinformatics command line tools and workflows for amplicon sequencing analysis, but requirements of bioinformatics skills often limit the efficient and up-to-date use of computational resources.

**Results:**

We present dadasnake, a user-friendly, 1-command Snakemake pipeline that wraps the preprocessing of sequencing reads and the delineation of exact sequence variants by using the favorably benchmarked and widely used DADA2 algorithm with a taxonomic classification and the post-processing of the resultant tables, including hand-off in standard formats. The suitability of the provided default configurations is demonstrated using mock community data from bacteria and archaea, as well as fungi.

**Conclusions:**

By use of Snakemake, dadasnake makes efficient use of high-performance computing infrastructures. Easy user configuration guarantees flexibility of all steps, including the processing of data from multiple sequencing platforms. It is easy to install dadasnake via conda environments. dadasnake is available at https://github.com/a-h-b/dadasnake.

## Findings

### Background

Since the first reports 15 years ago [1], high-throughput amplicon sequencing has become the most common approach to monitor microbial diversity in environmental samples. Sequencing preparation, throughput, and precision have been consistently improved, while costs have decreased. Computational methods have been refined in recent years, especially with the shift to exact sequence variants (ESVs = amplicon sequence variants, ASVs) and better use of sequence quality data [2, 3]. While amplicon sequencing can have severe limitations, such as limited and uneven taxonomic resolution [4, 5], over- and underestimation of diversity [6, 7], lack of absolute abundances [8,9], and missing functional information, amplicon sequencing is still considered the method of choice to gain an overview of microbial diversity and composition in a large number of samples [10, 11]. Consequently, the sizes of typical amplicon sequencing datasets have grown. In addition, synthesis efforts are undertaken, requiring efficient processing pipelines for amplicon sequencing data [12]. Owing to the unique, microbiome-specific characteristics of each dataset and the need to integrate the community structure data with other data types, such as abiotic or biotic parameters, users of data processing tools need to have expert knowledge on their biological question and statistics. It is therefore desirable that workflows be as user-friendly as possible. There are several widely used tool collections, e.g., QIIME 2 [13], mothur [14], usearch [15], and vsearch [16], and 1-stop pipelines, e.g., LotuS [17], with new approaches continually being developed, e.g., OCToPUS [18] and PEMA [19]. Typically, workflows balance learning curves, configurability, and efficiency.

### Purpose of dadasnake

dadasnake is a workflow for amplicon sequencing data processing into annotated ASVs. It is set up with microbial ecologists in mind, to be run on high-performance clusters without the users needing any expert knowledge on their operation. dadasnake is implemented in Snakemake [20] using the conda package management system. Consequently, it features a simple installation process, a 1-command execution, and high configurability of all steps with sensible defaults. dadasnake includes example workflows for common applications and produces a unique set of useful outputs, comprising relative abundance tables with taxonomic and other annotations in multiple formats, and reports on the data processing and visualizations of data quality at each step. The workflow is open-source, based on validated, favourably benchmarked tools.

### Implementation

**Figure 1:**
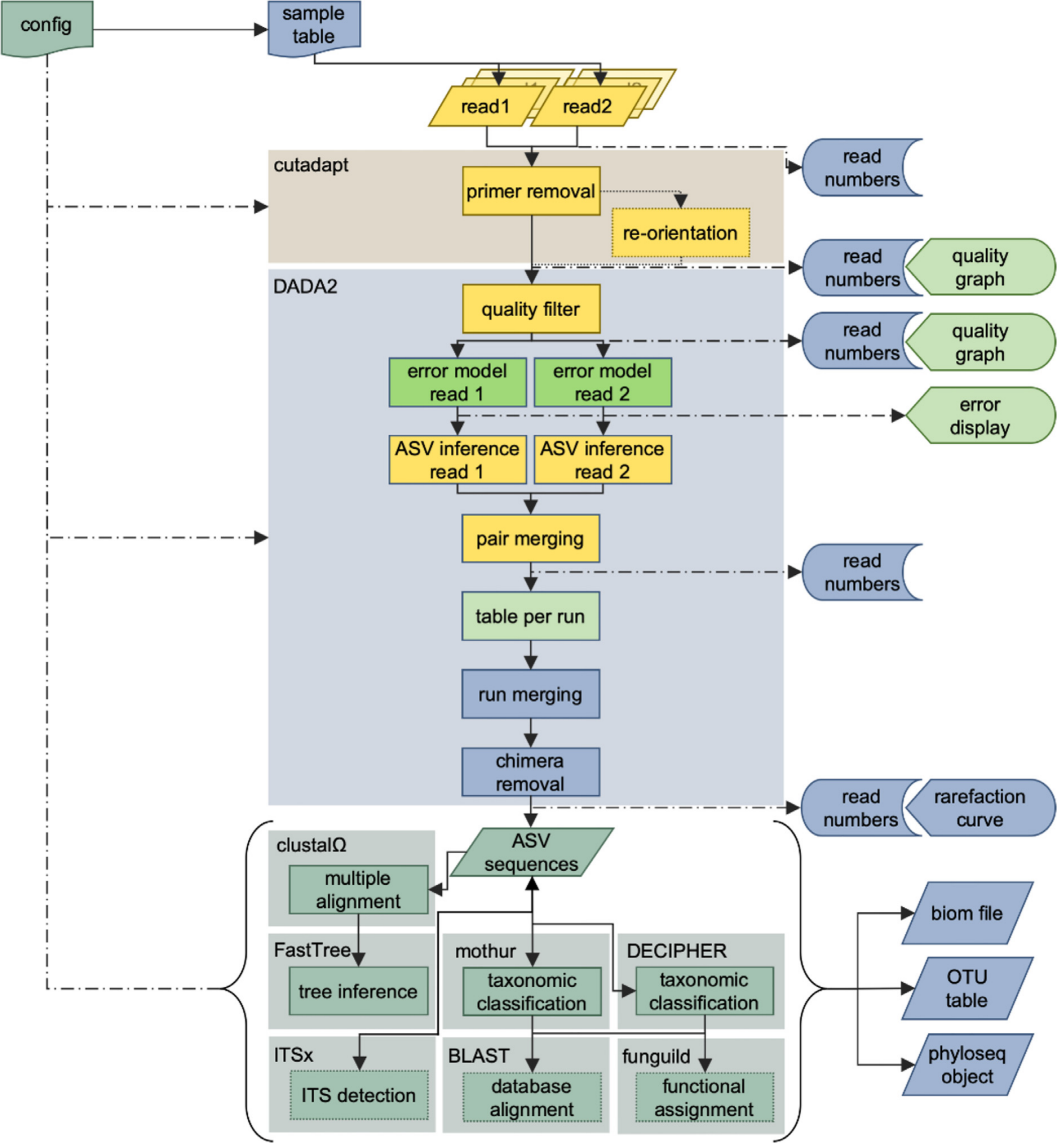
Overview of the dadasnake workflow for paired-end Illumina sequencing of a fungal ITS region with inputs (configuration file, sample table, and read files) and outputs (read numbers, graphical representations of quality and error models, rarefaction curves, and “OTU tables,” in biom, table, and phyloseq format). The steps are configurable and alternative workflows exist, e.g., for single-end, non-Illumina datasets, or other target regions. Primer removal and all post-DADA2 steps are optional. Colours represent the level of analysis: yellow: analysis per library/sample; bright green: analysis per run; sea green: analysis of the cumulated dataset; blue: analysis for the whole dataset with sample-wise documentation. Note that the DADA2 block can be performed in pooled mode at the level of the whole dataset.

The central processing within dadasnake wraps the DADA2 R package [21], which accurately determines sequence variants [22–24]. The dadasnake wrapper eases DADA2 use and deployment on computing clusters without the overhead of larger pipelines with DADA2 such as QIIME 2 [13]. Within dadasnake, the steps of quality filtering and trimming, error estimation, inference of sequence variants, and, optionally, chimera removal are performed (Fig. 1). Prior to quality filtering, dadasnake optionally removes primers and re-orients reads using cutadapt [25]. Taxonomic classification is realized using the reliable naive Bayes classifier as implemented in mothur [14] or DADA2, or by DECIPHER [26, 27] with optional species identification in DADA2. BLAST [28] can optionally be used to annotate all or only unclassified sequence variants. The sequence variants can be filtered on the basis of length, taxonomic classification, or recognizable regions, namely, by ITSx [29], before downstream analysis. For downstream analyses, a multiple alignment [30] and FastTree-generated tree [31] can be integrated into a phyloseq [32] object. Alternatively, tab-separated or R tables and standardized BIOM format [33] are generated. dadasnake records statistics, including numbers of reads passing each step, quality summaries, error models, and rarefaction curves [34]. All intermediate steps and configuration settings are saved for reproducibility.

Reproducibility, user-friendliness, and modular design are facilitated by the Snakemake framework, a popular workflow manager for reproducible and scalable data analyses (Snakemake, RRID:SCR_003475) [20]. Snakemake also generates HTML reports, which store code, version numbers, the workflow, and links to results. DADA2 and the other tools are packaged in conda environments to facilitate installation. For reasons of reproducibility, dadasnake uses fixed versions of all tools, which are regularly tested on mock datasets and updated when improvements become available. Snakemake also ensures flexible use as single-threaded local workflow or efficient deployment on a batch scheduling system. Currently slurm and univa/sun grid engine scheduler configurations are defined for dadasnake.

### dadasnake configuration and execution

The whole dadasnake workflow is started with a single command (“dadasnake -c configuration.yaml”). The user provides a tab-separated table with sample names and input files, as well as a configuration file in the simple, human-readable and -writable YAML format (see Supplementary File 1 for a worked example) to determine which steps should be taken and with what settings (see description of all configurable parameters in Supplementary Table 1). dadasnake is highly configurable compared with other Snakemake-based amplicon sequencing workflows, e.g., Hundo [35]. To facilitate its use, dadasnake provides easily adjustable, tested default settings and configuration files for several use cases.

dadasnake can use single-end or paired-end data. DADA2 can be efficiently used by parallelizing most steps by processing samples individually [36]. Pooled analysis can alternatively be chosen in dadasnake, and we recommend it for more error prone technologies such as 454 or third-generation long reads. While DADA2 has been designed for Illumina technology [21], dadasnake has been tested on Roche pyrosequencing data [37] and circular consensus Pacific Biosciences [38] and Oxford Nanopore data [39, 40] (see supporting material [60]). dadasnake provides example configurations for these technologies and for Illumina-based analysis of 16S, ITS, and 18S regions of bacterial and fungal communities.

dadasnake offers a range of different output formats for easy integration with downstream analysis tools. Tab-separated or R tables and standardized BIOM format [33], or a phyloseq [32] object are generated as final outputs in the user-defined output directory (see description of all outputs in Supplementary Table 2). Visualizations of the input read quality, read quality after filtering, the DADA2 error models, and rarefaction curves of the final dataset are also saved into a stats folder within the output. The numbers of reads passing each step are recorded for trouble-shooting. All intermediate steps and configuration settings are saved for reproducibility and to restart the workflow in case of problematic settings or datasets, so hard disk requirements are ∼1.3-fold the input data. The Snakemake-generated HTML report contains all software versions and settings to facilitate the publication of the workflow's results (see supporting material [60]).

Snakemake provides detailed error reports, and the logs of each step are recorded during runs. E-mail notifications of start and finishing can be sent. Users can find trouble-shooting help and file issues [41].

### Use cases: performance

To demonstrate dadasnake's performance, public datasets of different scales were processed. The performance of dadasnake depends strongly on the number of reads, number of samples, number of ASVs, and the required processing steps.

**Figure 2:**
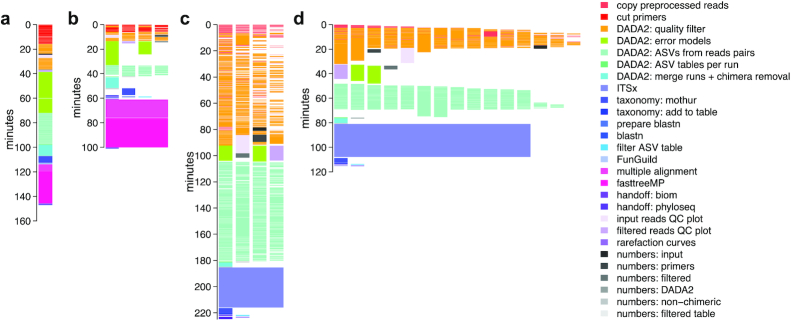
Visualization of resource use by processing different datasets. (a) The small (24 sample) 16S rRNA V4 amplicon dataset [42] processed linearly on a single core; (b) the same dataset processed on up to 4 cores (each depicted as a vertical stack); (c) a medium-sized (267 sample) ITS1 amplicon dataset [43], processed on up to 4 cores; (d) the same dataset, processed on up to 15 cores. Each block represents 1 job issued by dadasnake; colours represent the respective steps. QC: quality control.

Small datasets can be run on single cores with <8 GB RAM, but they profit from dadasnake's parallelization. For example, a 24-sample dataset with 2.9 million 16S ribosomal RNA (rRNA) V4 reads [42] could be completely processed, including preprocessing, quality filtering, ASV determination, taxonomic assignment, treeing, visualization of quality, and hand-off in various formats, with a total wall clock time of 150 minutes. Running time was reduced to 100 minutes, when 4 cores were used, especially owing to the parallelization of the preprocessing and ASV determination steps (Fig. 2a and b). Hardware requirements for small datasets are minimal, including small personal laptops. A medium-sized ITS1 dataset (267 samples with a total of 46.8 million reads [43]) could be processed in just under 4 hours on four 8 GB cores, including quality filtering, ASV determination, extraction of ITS1, taxonomic assignment, visualization of quality, and hand-off in various formats (Fig. 2c). While the system wall clock time was similar, the use of 15 cores reduced the runtime by a factor of 2 (Fig. 2d).

Generally speaking, dadasnake's parallelization of primer trimming, quality filtering, and ASV determination leads to shortened running times, while some steps, like merging of the ASV results of the single samples and all processing of assembled ASV tables, such as chimera removal, taxonomic annotation, and treeing, are run sequentially. While dadasnake requests more cores for steps that use parallelized tools, such as ITSx or treeing, the speed-up is usually incremental. Of note for users of shared cluster environments, dadasnake does not occupy cores idly; e.g., when only a single core is used for merging of runs and chimera removal (Fig. 2b–d) the other cores are available to other users, leading to high overall efficiency (>90%).

dadasnake is able to preprocess reads, report quality, determine ASVs, and assign taxonomy for very large datasets, e.g., the original 2.1 billion reads in >27,000 samples of the Earth Microbiome Project publication [12] within 87 real hours on only ≤50 CPU cores. Due to the independent handling of the preprocessing, filtering and ASV definition steps, the number of input samples only prolongs the run time linearly. Sample merging and handling of the final table, however, requires more RAM the more unique ASVs and samples are found (e.g., >190 GB for the >700,000 ASVs in the >27,000 samples of the Earth Microbiome Project). Tree building was not possible for this dataset on our infrastructure. For very large datasets it is therefore advisable to filter the final table before postprocessing steps.

### Use cases: accuracy

To demonstrate dadasnake's potential to accurately determine community composition and richness, two mock community datasets from Illumina sequencing of bacterial and archaean [44] and fungal [45] DNA were analysed (compositions displayed in Supplementary Table 3). In both cases, the genus-level composition was determined mostly correctly (Fig. 2a and b; Supplementary Table 3). One fungal taxon and 2 archaeal and 3 bacterial taxa were not detected at all, likely because they were not amplified. False-positive bacterial genera were unrelated to the taxa in the mock community and contained several human/skin-associated taxa, e.g., *Corynebacterium* and *Staphylococcus*, as well as commonly detected sequencing contaminants such as Rhizobiaceae and *Sphingomonas* (see overlap with [46] in Supplementary Table 3). The large number of false-positive results was therefore likely caused by contaminants in the bacterial dataset, which have been observed in this dataset before [24]. For the fungal dataset, 1 *Fusarium* sequence was misclassified as *Giberella*. In the same settings, the ASV richness was inferred close to correctly at 59 and 19 prokaryotic and fungal ASVs, respectively (ignoring the contaminants; Fig. 2c and d).

Next to accurate information on taxonomic composition and taxon richness, recognition of closely related strains is required from amplicon sequence processing tools. Six bacterial genera were represented by 2 strains each in the bacterial dataset and recognized as such by ASVs. In the case of 3 prokaryotic genera, the true diversity was not resolved by ASVs, with 3 *Thermotoga* strains and 2 *Salinispora* and 2 *Sulfitobacter* strains conflated as 2 and 1 strains, respectively (Supplementary Table 3). Micro-diversity was correctly identified for 2 strains of *Aspergillus* and the 3 *Fusarium strains* (although 1 was misclassified) for the fungal dataset. Strain diversity was overestimated for the fungal dataset in *Rhizophagus irregularis*, which is known to contain within-genome diversity of rRNA gene sequences [47]. Overall, dadasnake returns accurate results for taxonomic composition, richness, and micro-scale diversity within the limits of taxonomic resolution within short regions.

### Use cases: limitations

**Figure 3:**
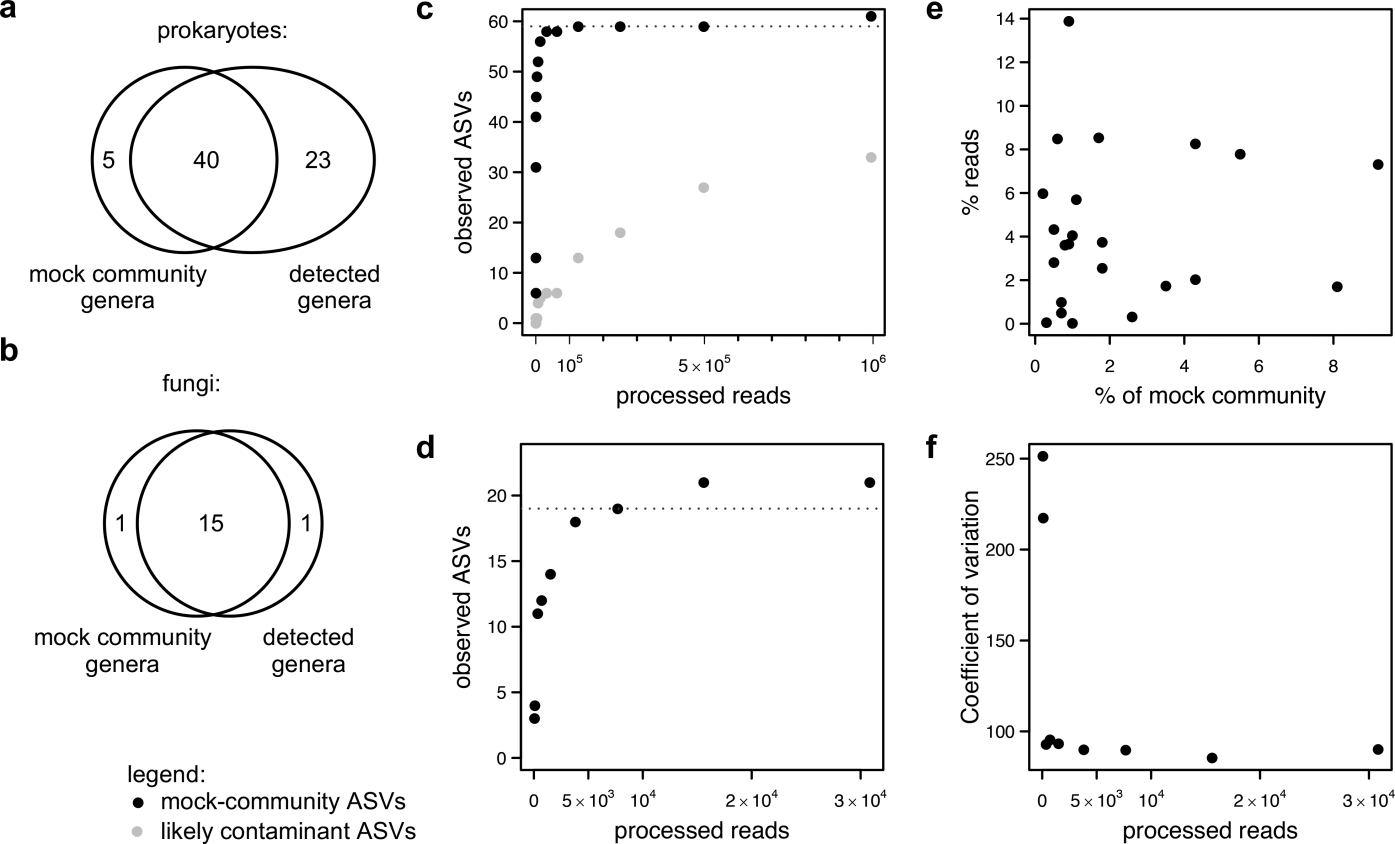
Comparison of mock community composition with analysis results. (a) Detection of prokaryotic genera at the highest sequencing depth (1.6 million reads); (b) detection of fungal genera at the highest sequencing depth (40,000 reads); (c) number of detected prokaryotic ASVs vs number of processed (non-chimeric) reads (black circles: ASVs of taxa from the mock community; grey circles: likely contaminant taxa); (d) number of detected fungal ASVs vs number of processed (non-chimeric) reads of the fungal mock community; (c, d) dotted lines indicate expected taxa richness; (e) missing correlation of real percentages of the mock communities and detected relative abundances of prokaryotic genera; (f) coefficients of variation between relative abundances of taxa that should be equally abundant in the fungal mock community.

The analysis of the mock community data also revealed limitations of the approach in general. A commonly used approach to detect underestimation of richness at low sequencing depths is to plot rarefaction curves or use richness estimators [48–50], which use subsamples of the assigned reads to model how much the addition of further sequencing would increase the observed richness. However, the statistical requirements for delineation of ASVs mean that not all sequenced taxa are represented by an ASV in a given data set [51]. This in turn leads to the flattening of rarefaction curves derived from finished ASV tables, although an increase in real sequencing depth would lead to a greater number of observed ASVs (Fig. 3c and d). Richness estimates and rarefaction curves based on DADA2 datasets need to be handled with caution and, whenever richness estimates are essential, should be based on subsamples that are processed by DADA2 independently rather than post hoc models.

A second limitation, common to amplicon sequencing, is that relative abundances of ASVs are not reflective of the actual abundance of the sequenced taxa, which varied for the prokaryotic mock community and were equal in the fungal mock community. Specifically, the relative abundance of the prokaryotic taxa did not correlate with the relative abundance of reads (Fig. 2e). The relative abundance of reads for the fungal taxa varied by several orders of magnitude, despite equal inputs (Fig. 3f). There are numerous reasons for misrepresentation of abundances by PCR-based analyses [52]. Of note, the variation in the relative abundance estimates is observed to be highest at low sequencing depths (Fig. 3e and f). Therefore, whenever comparisons of relative abundances within samples are undertaken, it is necessary to, at the least, ensure that sequencing depths of all samples are sufficient to reach stable estimates. However, the analysis of the mock community case studies also suggests that true relative abundances can never be determined, which should be accounted for in experimental design and interpretation.

## Methods

### Bacterial and archaean mock community dataset

The largest library of the Illumina sequencing datasets of a 59-species mock community [53], comprising 10 archaea and 49 bacteria (for composition see Supplementary Table 3), was retrieved from the European Nucleotide Archive (ENA) under accession ERR777696. The ground-truth composition of the mock community was manually extracted from the publication and the taxonomic names adapted to the convention of the SILVA v. 138 database [54]. To analyse the effect of sequencing depth on the recovery of the mock community, the dataset was subsampled to 100, 200, 500, 1,000, 2,000, 5,000, 10,000, 20,000, 50,000, 100,000, 200,000, 400,000, 800,000, and 1,600,000 read pairs.

The same configuration was used to run dadasnake on all subsamples. The most important settings include removal of the primers from either read (515F, specified as 5-GTGYCAGCMGCCGCGGTAA, and 806R, specified as 5-GGACTACNVGGGTWTCTAAT, with a maximum of 20% mismatch); truncation of the reads at positions with a quality <13, before removal of forward and reverse reads with <170 and 130 nucleotide length, respectively, and truncation to these lengths before removal of reads with an expected error >0.2; requirement of a minimum of 12 bp overlap for merging of denoised sequences; and removal of chimeras on consensus.

### Fungal mock community sequencing

The ITS2 region of an even (i.e. having equal proportions of each species) 19-species fungal mock community [45] provided by Matt Bakker (U.S. Department of Agriculture, Peoria, IL, US) for composition see Supplementary Table 3) was amplified using the primers F-ITS4 5-TCCTCCGCTTATTGATATGC [55] and R-fITS7 5-GTGARTCATCGAATCTTTG [56] modified with heterogeneity spacers according to Cruaud et al. [57]. Amplicon libraries were prepared using the Nextera XT kit (Illumina) and sequenced on an Illumina MiSeq (Illumina MiSeq System, RRID:SCR_016379) with v.3 chemistry at 2 × 300 bp. Sequencing was performed in triplicate, and all reads were pooled for the analysis presented here. The sequencing data are accessible at the NCBI SRA under BioProject accession PRJNA626434. The ground-truth composition of the data was manually extracted from the publication and the taxonomic names were adjusted to the ones used in the Unite 8.0 database. To analyse the effect of sequencing depth on the recovery of the mock community, the dataset was subsampled to 100, 200, 500, 1,000, 2,000, 5,000, 10,000, 20,000, and 40,000 reads.

The same configuration was used for running dadasnake on all subsamples. The most important settings were as follows: removal of the primers from either read with a maximum of 20% mismatch; truncation of the reads at positions with a quality <15, before removal of reads with <70 nucleotide length and removal of reads with an expected error >3; requirement of a minimum of 20 bp overlap for merging of denoised sequences; removal of chimeras on consensus; and ITSx was run on the ASVs, which would remove non-fungal ASVs (which did not occur in the mock community).

### Performance testing

To demonstrate dadasnake's performance on a small laptop computer, a small dataset of 24 16S rRNA gene amplicon sequences from a local soil fertilization study [42] were downloaded from the NCBI SRA (PRJNA517390) using the fastq-dump function of the SRA-toolkit. Using the settings optimized for the bacterial mock community, dadasnake was run either on a computer cluster using 1 or ≤4 threads with 8 GB RAM each, or without cluster-mode on 3 cores of a laptop with an Intel i5-2520M CPU with 2.5 GHz and 8 GB shared RAM.

To compare the performance of dadasnake on a medium-sized study in different settings, ITS1 amplicon sequences of 267 samples measured using Illumina HiSeq technology in a global study on fertilization effects [43] were downloaded from the NCBI SRA (PRJNA272747) using the fastq-dump function of the SRA-toolkit. Owing to the variable length of the ITS1 region, reads were not truncated to a specified length but trimmed to a minimum per-base quality of 15 (also discarding reads with a maximum expected error >3). After error modelling and ASV construction per sample, read pairs were merged with ≥20 bp overlap, allowing for 2 mismatches. After table set-up, the ITSx classifier was run to remove non-fungal ASVs before taxonomic annotation (using the mothur [14] classifier; for configuration see Supplementary File 1). The same runs were performed on either a compute cluster using ≤50 threads or only ≤4 threads with 8 GB RAM each.

A total of 27,081 samples analysed by the Earth Microbiome Project [12] stored under accessions ERP021896, ERP020023, ERP020508, ERP017166, ERP020507, ERP017221, ERP016412, ERP020884, ERP020022, ERP020510, ERP017438, ERP016395, ERP020539, ERP016468, ERP020590, ERP020021, ERP020587, ERP020560, ERP020589, ERP017176, ERP017220, ERP017174, ERP016405, ERP020591, ERP021691, ERP016416, ERP022167, ERP021699, ERP016495, ERP022245, ERP016748, ERP016749, ERP016752, ERP016540, ERP006348, ERP016543, ERP016746, ERP016586, ERP016735, ERP021864, ERP016588, ERP016587, ERP016539, ERP016734, ERP016492, ERP003782, ERP016607, ERP016581, ERP016557, ERP016464, ERP016542, ERP016541, ERP016591, ERP016854, ERP016852, ERP016286, ERP016451, ERP023684, ERP016869, ERP010098, ERP016879, ERP016883, ERP016466, ERP016496, ERP016880, ERP016455, ERP016900, ERP016924, ERP016923, ERP016925, ERP016927, ERP016469, ERP016329, ERP016926, ERP021540, ERP021541, ERP021542, ERP021543, ERP021544, ERP021545, ERP016937, ERP016131, ERP016483, ERP016252, ERP022166, ERP016414, ERP016472, ERP023686, ERP017459, ERP016287, ERP016285, ERP005806, ERP021895, ERP016384, ERP016491, and ERP006348 were downloaded from the NCBI SRA using the fastq-dump function of the SRA-toolkit. In accordance with the published analysis, reads were trimmed to 90 bp, before quality control (discarding reads with a maximum expected error >0.2 or positions with <13 quality score), error modelling (per project accession), ASV construction (per sample), table set-up, and taxonomic annotation (using the mothur [14] classifier). To handle the combined dataset table, 360 GB RAM were reserved for the final steps in R.

Efficiency was calculated as the ratio of CPU time divided by the product of slots used and real wall clock time.

## Databases

The SILVA [54] RefSSU_NR99 database v. 138 was used for the taxonomic classification of bacterial and archaean ASVs. Fungal ASVs were classified against the UNITE v8 database [58, 59]. Both sets of ASVs were classified using the Bayesian classifier as implemented in mothur's classify.seqs command [14], with a cut-off of 60.

## Visualization and Statistics

The output of all dadasnake runs was gathered in an R-workspace (for tabular version see Supplementary Table 3). Rarefaction curves were plotted using vegan [34]. The coefficient of variation was calculated as the ratio of the standard deviation to the mean. The cluster-job information for the performance tests was gathered in an R-workspace. Efficiency was calculated as the ratio of CPU time divided by the product of slots used and real wall clock time.

## Availability of Supporting Source Code and Requirements

Project name: dadasnake

Project home page: https://github.com/a-h-b/dadasnake

Operating system: Linux

Programming language: Python, R, bash

Other requirements: anaconda or other conda package manager

License: GNU GPL-3.0

RRID:SCR_019149

## Data Availability

The raw sequencing data generated for this article are accessible on NCBI's SRA under BioProject accession PRJNA626434. Processing results of the mock community datasets, the ground-truth mock community compositions, and the scripts to visualize the use case datasets are available from Zenodo [60]. The frozen version of dadasnake described in this article is available from Zenodo [61].

## Additional Files

**Supplementary File 1:** Example of a YAML configuration file: configuration for the large dataset of the performance test.

**Supplementary Table 1:** Description of all configurable settings.

**Supplementary Table 2:** Description of outputs.

**Supplementary Table 3:** Mock community compositions and identification of ASVs from mock community datasets.

## Abbreviations

ASV: amplicon sequence variant; BIOM: Biological Observation Matrix; BLAST: Basic Local Alignment Search Tool; bp: base pairs; CPU: central processing unit; ESV: exact sequence variant; ITS: internal transcribed spacer; NCBI: National Center for Biotechnology Information; OCToPUS: Optimized CATCh, mothur, IPED, UPARSE, and SPAdes; OTU: operational taxonomic unit; PEMA: Pipeline for Environmental DNA Metabarcoding Analysis; QIIME: Quantitative Insights Into Microbial Ecology; RAM: random access memory; rRNA: ribosomal RNA; SRA: Sequence Read Archive.

## Competing Interests

The authors declare that they have no competing interests.

## Funding

A.H.-B. was funded by the German Centre for Integrative Biodiversity Research (iDiv) Halle-Jena-Leipzig of the German Research Foundation (DFG - FZT118, grant No. 202548816). C.W. acknowledges funding from the German Research Foundation (DFG - GFBio II, grant No. BU 941/23-2).

## Authors' Contributions

Conceptualization, software, analysis, writing: A.H.-B.; optimization and testing: C.W.; sequencing: B.S. All authors contributed to the manuscript text and approved its contents.

## Supplementary Material

giaa135_GIGA-D-20-00147_Original_Submission

giaa135_GIGA-D-20-00147_Revision_1

giaa135_Response_to_Reviewer_Comments_Original_Submission

giaa135_Reviewer_1_Report_Original_Submission Falk Hildebrand -- 6/13/2020 Reviewed

giaa135_Reviewer_2_Report_Original_Submission Benjamin Callahan -- 6/19/2020 Reviewed

giaa135_Reviewer_2_Report_Revision_1 Benjamin Callahan -- 10/26/2020 Reviewed

giaa135_Supplemental_Files
