# Supplementary material for: Dadasnake, a Snakemake implementation of DADA2 to process amplicon sequencing data for microbial ecology
Source: Gigascience. 2020 Nov 30;9(12):giaa135. doi: 10.1093/gigascience/giaa135 (PMC7702218; doi:10.1093/gigascience/giaa135)
Supplement: giaa135_GIGA-D-20-00147_Original_Submission [file giaa135_giga-d-20-00147_original_submission.pdf]

GigaScience

Efficiently processing amplicon sequencing data for microbial ecology with dadasnake, DADA2 in Snakemake

--Manuscript Draft--

**Manuscript Number:**	GIGA-D-20-00147
**Full Title:**	Efficiently processing amplicon sequencing data for microbial ecology with dadasnake, DADA2 in Snakemake
**Article Type:**	Technical Note
**Funding Information:**	Deutsche Forschungsgemeinschaft (FZT118) — Not applicable
**Abstract:**	Background: Amplicon sequencing of phylogenetic marker genes, e.g. 16S, 18S or ITS rRNA sequences, is still the most commonly used method to estimate the structure of microbial communities. Microbial ecologists often have expert knowledge on their biological question and data analysis in general, and most research institutes have computational infrastructures to employ the bioinformatics command line tools and workflows for amplicon sequencing analysis, but requirements of bioinformatics skills often limit the efficient and up-to-date use of computational resources.<br>Results: dadasnake wraps pre-processing of sequencing reads, delineation of exact sequencing variants using the favorably benchmarked, widely-used the DADA2 algorithm, taxonomic classification and post-processing of the resultant tables, and hand-off in standard formats, into a user-friendly, one-command Snakemake pipeline. The suitability of the provided default configurations is demonstrated using mock-community data from bacteria and archaea, as well as fungi.<br>Conclusions: By use of Snakemake, dadasnake makes efficient use of high-performance computing infrastructures. Easy user configuration guarantees flexibility of all steps, including the processing of data from multiple sequencing platforms. dadasnake facilitates easy installation via conda environments. dadasnake is available at https://github.com/a-h-b/dadasnake .
**Corresponding Author:**	Anna Heintz-Buschart<br>Helmholtz-Zentrum fur Umweltforschung UFZ<br>Halle, GERMANY
**Corresponding Author Secondary Information:**	
**Corresponding Author's Institution:**	Helmholtz-Zentrum fur Umweltforschung UFZ
**Corresponding Author's Secondary Institution:**	
**First Author:**	Christina Weißbecker
**First Author Secondary Information:**	
**Order of Authors:**	Christina Weißbecker
	Beatrix Schnabel
	Anna Heintz-Buschart
**Order of Authors Secondary Information:**	
**Additional Information:**	
**Question**	**Response**
Are you submitting this manuscript to a special series or article collection?	No
**Experimental design and statistics**	Yes

Powered by Editorial Manager® and ProduXion Manager® from Aries Systems Corporation

Full details of the experimental design and statistical methods used should be given in the Methods section, as detailed in our Minimum Standards Reporting Checklist. Information essential to interpreting the data presented should be made available in the figure legends.<br><br>Have you included all the information requested in your manuscript?	
**Resources**<br><br>A description of all resources used, including antibodies, cell lines, animals and software tools, with enough information to allow them to be uniquely identified, should be included in the Methods section. Authors are strongly encouraged to cite Research Resource Identifiers (RRIDs) for antibodies, model organisms and tools, where possible.<br><br>Have you included the information requested as detailed in our Minimum Standards Reporting Checklist?	Yes
**Availability of data and materials**<br><br>All datasets and code on which the conclusions of the paper rely must be either included in your submission or deposited in publicly available repositories (where available and ethically appropriate), referencing such data using a unique identifier in the references and in the "Availability of Data and Materials" section of your manuscript.<br><br>Have you have met the above requirement as detailed in our Minimum Standards Reporting Checklist?	Yes

Powered by Editorial Manager® and ProduXion Manager® from Aries Systems Corporation

Manuscript Click here to download Manuscript 200518_no_fig.pdf

Efficiently processing amplicon sequencing data for microbial ecology with dadasnake, DADA2 in Snakemake

Christina Weißbecker[1], *Beatrix Schnabel*[1], *Anna Heintz-Buschart*[1,2]*

*corresponding author, email: anna.heintz-buschart@ufz.de

[1]Helmholtz Centre for Environmental Research GmbH - UFZ, Department of Soil Ecology

[2]German Centre for Integrative Biodiversity Research (iDiv) Halle-Jena-Leipzig, Bioinformatics Unit

Abstract

Background: Amplicon sequencing of phylogenetic marker genes, e.g. 16S, 18S or ITS rRNA sequences, is still the most commonly used method to estimate the structure of microbial communities. Microbial ecologists often have expert knowledge on their biological question and data analysis in general, and most research institutes have computational infrastructures to employ the bioinformatics command line tools and workflows for amplicon sequencing analysis, but requirements of bioinformatics skills often limit the efficient and up-to-date use of computational resources.
Results: dadasnake wraps pre-processing of sequencing reads, delineation of exact sequencing variants using the favorably benchmarked, widely-used the DADA2 algorithm, taxonomic classification and post-processing of the resultant tables, and hand-off in standard formats, into a user-friendly, one-command Snakemake pipeline. The suitability of the provided default configurations is demonstrated using mock-community data from bacteria and archaea, as well as fungi.
Conclusions: By use of Snakemake, dadasnake makes efficient use of high-performance computing infrastructures. Easy user configuration guarantees flexibility of all steps, including the processing of data from multiple sequencing platforms. dadasnake facilitates easy installation via conda environments. dadasnake is available at https://github.com/a-h-b/dadasnake .

Keywords
rRNA gene sequence analysis; denoising; exact sequence variants; R; pipeline; microbiome; community structure

Findings

Background

Since the first reports 15 years ago [1], high-throughput amplicon sequencing has become the most common approach to monitor microbial diversity in environmental samples. Sequencing preparation, throughput and precision have been consistently improved, while costs have decreased. Computational methods have been refined in the recent years, especially with the shift to exact sequencing variants and better use of sequence quality data [2,3]. While amplicon sequencing can have severe limitations, such as limited and uneven taxonomic resolution [4,5], over- and underestimation of diversity [6,7], lack of quantitative value [8,9] and missing functional information, amplicon sequencing is still considered the method of choice to gain an overview of microbial diversity in a large number of samples [10,11]. Consequently, the sizes of typical amplicon sequencing datasets have grown. In addition, synthesis efforts are undertaken, requiring efficient processing pipelines for amplicon sequencing data [12]. Due to the unique, microbiome-specific characteristics of each dataset and the need to integrate the community structure data with other data types, such as abiotic or biotic parameters, users of data processing tools need to have expert knowledge on their biological question and statistics. It is therefore desirable that workflows should be as user-friendly as possible. Several widely used workflows exist e.g. qiime2 [13], mothur [14], usearch [15], lOTUs [16], with new approaches continually being developed, e.g. OCToPUS [17], PEMA [18], typically balancing learning curves, configurability and efficiency.

Purpose of dadasnake

dadasnake is a workflow for amplicon sequence processing that is set up with microbial ecologists in mind, to be run on high-performance clusters without the users needing any expert knowledge on their operation. dadasnake is implemented in Snakemake [19] using the conda package management system. Consequently, it features a simple installation process, a one-command execution, high configurability of all steps, with sensible defaults and example workflows for common applications, and hand-off to popular R and biom formats. The workflow is open-source, based on validated, favourably benchmarked tools.

Implementation

The central processing within dadasnake wraps the DADA2 R package [20], which accurately determines sequence variants [21-23]. Within dadasnake, the steps of quality filtering and trimming, error estimation, inference of sequence variants, and, optionally, chimera removal are performed (Figure 1). Prior to quality filtering, dadasnake optionally removes primers and re-orients reads using cutadapt [24]. Taxonomic classification is realized using the reliable naïve Bayes classifier as implemented in mothur [14], or by DECIPHER [25,26] with optional species-identification in DADA2. The sequencing variants can be filtered based on length, taxonomic classification or recognizable regions, namely ITSx [27] before downstream analysis. For downstream analyses, a multiple alignment [28] and FastTree-generated tree [29] can be integrated into a phyloseq [30] object. Alternatively, tab-separated or R tables and standardized BIOM format (https://biom-format.org/index.html) are generated. dadasnake records statistics, including numbers of reads passing each step, quality summaries, error models, and rarefaction curves [31]. All intermediary steps and configuration settings are saved for reproducibility.
Reproducibility, user-friendliness and modular design are facilitated by the Snakemake framework [19]. An html report can be generated for each run, which stores code, version numbers, the workflow and links to results. DADA2 and the other tools are packaged in a conda environment to facilitate installation. Snakemake also ensures flexible use as single-threaded local workflow or efficient deployment on a batch scheduling system. Currently slurm and univa/sun grid engine scheduler configurations are defined.

Configuration

At run time, the user provides a tab-separated table with sample names and files, as well as a configuration file, which determines which steps should be taken and with what settings (see supplementary table 1). dadasnake can use single end or paired end data. DADA2 can be efficiently employed by parallelizing most steps by processing samples individually (https://benjjneb.github.io/dada2/bigdata.html). Pooled analysis can alternatively be chosen in

dadasnake and is recommended for more error prone technologies such as 454 or third generation long reads. While DADA2 has been designed for Illumina technology [20], dadasnake has been tested on Roche pyrosequencing data [32] and circular consensus Pacbio [33] and Oxford Nanopore data [34,35]. dadasnake provides example configurations for these technologies and for Illumina-based analysis of 16S, ITS and 18S regions of bacterial and fungal communities.

Use case

To demonstrate dadasnake's potential to accurately determine community composition and richness, two mock community datasets from Illumina sequencing of bacterial [36] and fungal [37] DNA were analysed. In both cases, the genus-level composition was determined mostly correctly (Figure 2 a&b; supplementary table 2). One fungal and five bacterial taxa of the two mock communities were not detected at all, likely because they were not amplified. False positive bacterial genera were likely caused by contaminations in the bacterial dataset which have been observed in this dataset before [23]. For the fungal dataset, one *Fusarium* sequence was misclassified as *Giberella.* In the same settings, the ASV richness was inferred close to correctly at 59 and 19 prokaryotic and fungal ASVs, respectively (ignoring the contaminants; Figure 2 c&d).

Next to accurate information on taxonomic composition and taxon richness, recognition of closely related strains is required from amplicon sequence processing tools. Six bacterial genera were represented by two strains each in the bacterial dataset and recognized as such by ASVs. In the case of three prokaryotic genera, the true diversity was not resolved by ASVs, with three *Thermotoga* strains and two *Salinispora* and two *Sulfitobacter* strains conflated as two and one strains, respectively (supplementary table 2). Micro-diversity was correctly identified for two strains of *Aspergillus* and the three *Fusarium strains* (although one was misclassified) for the fungal dataset. Strain-diversity was overestimated for the fungal dataset in *Rhizophagus irregularis,* which is known to contain within-genome diversity of ribosomal RNA gene sequences [38]. Overall, dadasnake returns accurate results for taxonomic composition, richness and micro-scale diversity within the limits of taxonomic resolution within short regions.

Limitations

The analysis of the mock community data also revealed limitations. At low sequencing depths, the sample richness is prone to be underestimated (Figure 2 c&d). A commonly used approach to detect underestimation is to plot rarefaction curves or use richness estimators [39-41], which use subsamples of the assigned reads to model how much the addition of further sequencing would increase the observed richness. However, the statistical requirements for delineation of ASVs mean that not all sequenced taxa are represented by an ASV in a given data set. This in turn leads to the flattening of rarefaction curves derived from finished ASV tables (Figure 3 a&b), although an increase in real sequencing depth would lead to a greater number of observed ASVs (Figure 2 c&d). Richness estimates and rarefaction curves based on DADA2 datasets need to be handled with caution and whenever richness estimates are essential should be based on subsamples that are processed by DADA2 independently rather than post-hoc models.

A second limitation is that relative abundances of ASVs are not reflective of the actual abundance of the sequenced taxa, which varied for the prokaryotic mock community and were equal in the fungal mock community (Figure 3 c&d). There are numerous reasons for misrepresentation of abundances by PCR-based analyses [8,42]. Of note, the variation in the relative abundance estimates is observed to be highest at low sequencing depths (Figure 3 c&d). Therefore, whenever comparisons of relative abundances within samples are undertaken, it is necessary to, at the least, ensure that sequencing depths of all samples are sufficient to reach stable estimates. However, the analysis of the mock community case studies also suggests that true relative abundances can never be determined, which should be accounted for in experimental design and interpretation.

Methods

Bacterial and archaean mock community data set

The largest library of the Illumina sequencing datasets of a 59 species mock-community [43] was retrieved from the European Nucleotide Archive ENA under accession ERR777696. The ground-truth composition of the mock-community was manually extracted from the publication and the taxonomic

names adapted to the convention of the SILVA v. 138 database [44]. To analyse the effect of sequencing depth on the recovery of the mock-community, the dataset was subsampled to 100, 200, 500, 1,000, 2,000, 5,000, 10,000, 20,000, 50,000, 100,000, 200,000, 400,000, 800,000, and 1,600,000 read pairs.
The same configuration was used to run dadasnake on all subsamples. The most important settings include removal of the primers from either read (515F, specified as 5-GTGYCAGCMGCCGCGGTAA, and 806R, specified as 5-GGACTACNVGGGTWTCTAAT, with a maximum of 20 % mismatch); truncation of the reads at positions with a quality below 13, before removal of forward and reverse reads with less than 170 and 130 nt length, respectively, and truncation to these lengths before removal of reads with an expected error above 0.2; a minimum of 12 bp overlap was required for merging of denoised sequences; chimeras were removed on consensus.

Fungal mock community sequencing
The ITS2 region of a fungal mock community [37] was amplified using the primers F-ITS4 5-TCCTCCGCTTATTGATATGC [45] and R-fITS7 5-GTGARTCATCGAATCTTTG [46] modified with heterogeneity spacers according to [47]. Amplicon libraries were prepared using the Nextera XT kit (Illumina) and sequenced on an Illumina MiSeq with v.3 chemistry at 2 x 300 bp. Sequencing was performed in triplicates and all reads were pooled for the analysis presented here. The sequencing data is accessible at the NCBI Short Read Archive under BioProject accession PRJNA626434. The ground-truth composition of the data was manually extracted from the publication and the taxonomic names were adjusted to the ones used in the Unite 8.0 database. To analyse the effect of sequencing depth on the recovers of the mock-community, the dataset was subsampled to 100, 200, 500, 1,000, 2,000, 5,000, 10,000, 20,000 and 40,000 reads.
The same configuration was used for running dadasnake on all subsamples. The most important settings were: removal of the primers from either read with a maximum of 20 % mismatch; truncation of the reads at positions with a quality below 15, before removal of reads with less than 70 nt length and removal of reads with an expected error above 3; a minimum of 20 bp overlap was required for merging of denoised sequences; chimeras were removed on consensus.

Databases
The SILVA [44] RefSSU_NR99 database v. 138 was used for the taxonomic classification of bacterial and archaean ASVs. Fungal ASVs were classified against the UNITE v8 database [48,49]. Both sets of ASVs were classified using the Bayesian classifier as implemented in mothur's classify.seqs command [14], with a cut-off of 60.

Visualization
The output of all dadasnake runs was gathered in an R-workspace (tabular version see supplementary table 2). Rarefaction curves were plotted using vegan [31].

Availability of supporting source code and requirements
Project name: dadasnake
Project home page: https://github.com/a-h-b/dadasnake
Operating system(s): Linux
Programming language: Python, R, bash
Other requirements: anaconda or other conda package manager
License: GNU GPL-3.0

Availability of supporting data
The raw sequencing data generated for this manuscript are accessible on NCBI's Sequence Read Archive under BioProject accession PRJNA626434. Processing results of the mock community data sets, the ground-truth mock community compositions, and the scripts to visualize the use case datasets are available from Zenodo http://doi.org/10.5281/zenodo.3826697 .

List of abbreviations
ASV – amplicon sequencing variants (=ESV)

bp – basepairs
DADA2 – divisive amplicon denoising algorithm (version 2)
ESV – exact sequencing variants (=ASV)
ITS – intergenic transcribed spacer region of the rRNA genes operon
nt – nucleotides
OTU – operational taxonomic unit
rRNA – ribosomal RNA

Competing interests

The authors declare that they have no competing interests.

Funding

AH-B was funded by the German Centre for Integrative Biodiversity Research (iDiv) Halle-Jena-Leipzig of the German Research Foundation, FZT118.

Authors' contributions

Conceptualization, software, analysis, writing: AH-B; optimization and testing: CW; sequencing: BS. All authors contributed to the manuscript text and approve its contents.

Acknowledgements

The authors would like to acknowledge Kezia Goldmann and Julia Moll for testing early versions of the workflow; François Buscot for funding acquisition and providing resources; Guillaume Lentendu for discussions. Data processing has been performed at the High-Performance Computing (HPC) Cluster EVE, a joint effort of both the Helmholtz Centre for Environmental Research - UFZ and the German Centre for Integrative Biodiversity Research (iDiv) Halle-Jena-Leipzig and the authors thank Christian Krause and the other administrators for excellent support. Matthew Bakker is acknowledged for the generous provision of the fungal mock community.

References

1. Sogin ML, Morrison HG, Huber JA, Mark Welch D, Huse SM, Neal PR, et al. Microbial diversity in the deep sea and the underexplored "rare biosphere". Proc Natl Acad Sci USA. 2006;103:12115–20.

2. Callahan BJ, McMurdie PJ, Holmes SP. Exact sequence variants should replace operational taxonomic units in marker-gene data analysis. The ISME Journal. Nature Publishing Group; 2017;11:2639–43.

3. Glassman SI, Martiny JB. Ecological patterns are robust to use of exact sequence variants versus operational taxonomic units. 2018;:1–25.

4. Johnson JS, Spakowicz DJ, Hong B-Y, Petersen LM, Demkowicz P, Chen L, et al. Evaluation of 16S rRNA gene sequencing for species and strain-level microbiome analysis. Nature Communications. 2019;10:5029.

5. Brumfield KD, Huq A, Colwell RR, Olds JL, Leddy MB. Microbial resolution of whole genome shotgun and 16S amplicon metagenomic sequencing using publicly available NEON data. Gyarmati P, editor. PLoS ONE. Public Library of Science; 2020;15:e0228899–21.

6. Hugerth LW, Andersson AF. Analysing microbial community composition through amplicon eequencing: from aampling to hypothesis testing. Front. Microbiol. 2017;8:23–22.

7. Nearing JT, Douglas GM, Comeau AM, Langille MGI. Denoising the Denoisers: an independent evaluation of microbiome sequence error-correction approaches. PeerJ. PeerJ Inc; 2018;6:e5364–22.

8. Piwosz K, Shabarova T, Pernthaler J, Posch T, Simek K, Porcal P, et al. Bacterial and eukaryotic small-subunit amplicon data do not provide a quantitative picture of microbial communities, but they

are reliable in the context of ecological interpretations. McMahon K, editor. mSphere. American Society for Microbiology Journals; 2020;5:66–14.

9. Yeh Y-C, Needham DM, Sieradzki ET, Fuhrman JA. Taxon disappearance from microbiome analysis reinforces the value of mock communities as a standard in every sequencing run. Caporaso JG, editor. mSystems. American Society for Microbiology Journals; 2018;3:337–9.

10. Tessler M, Neumann JS, Afshinnekoo E, Pineda M, Hersch R, Velho LFM, et al. Large-scale differences in microbial biodiversity discovery between 16S amplicon and shotgun sequencing. Scientific Reports. Nature Publishing Group; 2017;7:6589–14.

11. Rausch P, Rühlemann M, Hermes BM, Doms S, Dagan T, Dierking K, et al. Comparative analysis of amplicon and metagenomic sequencing methods reveals key features in the evolution of animal metaorganisms. Microbiome. BioMed Central; 2019;7:133–19.

12. Thompson LR, Sanders JG, McDonald D, Amir A, Ladau J, Locey KJ, et al. A communal catalogue reveals Earth’s multiscale microbial diversity. Nature. Nature Publishing Group; 2017;551:457–63.

13. Bolyen E, Rideout JR, Dillon MR, Bokulich NA, Abnet CC, Al-Ghalith GA, et al. Reproducible, interactive, scalable and extensible microbiome data science using QIIME 2. Nat. Biotechnol. Nature Publishing Group; 2019;37:852–7.

14. Schloss PD, Westcott SL, Ryabin T, Hall JR, Hartmann M, Hollister EB, et al. Introducing mothur: open-source, platform-independent, community-supported software for describing and comparing microbial communities. Applied and Environmental Microbiology. 2009;75:7537–41.

15. Edgar RC. UPARSE: highly accurate OTU sequences from microbial amplicon reads. Nat Meth. 2013;10:996–8.

16. Hildebrand F, Tadeo R, Voigt AY, Bork P, Raes J. LotuS: an efficient and user-friendly OTU processing pipeline. Microbiome. 2014;2:30.

17. Mysara M, Njima M, Leys N, Raes J, Monsieurs P. From reads to operational taxonomic units: an ensemble processing pipeline for MiSeq amplicon sequencing data. GigaScience. 2017;6:1–10.

18. Zafeiropoulos H, Viet HQ, Vasileiadou K, Potirakis A, Arvanitidis C, Topalis P, et al. PEMA: a flexible Pipeline for Environmental DNA Metabarcoding Analysis of the 16S/18S ribosomal RNA, ITS, and COI marker genes. GigaScience. Oxford University Press; 2020;9:111–2.

19. Köster J, Rahmann S. Snakemake - a scalable bioinformatics workflow engine. Bioinformatics. 2012;28:2520–2.

20. Callahan BJ, McMurdie PJ, Rosen MJ, Han AW, Johnson AJA, Holmes SP. DADA2: High-resolution sample inference from Illumina amplicon data. Nat. Methods. 2016;13:581–3.

21. Pauvert C, Buée M, Laval V, Edel-Hermann V, Fauchery L, Gautier A, et al. Bioinformatics matters: The accuracy of plant and soil fungal community data is highly dependent on the metabarcoding pipeline. Fungal Ecology. Elsevier Ltd; 2019;41:23–33.

22. Caruso V, Song X, Asquith M, Karstens L. Performance of microbiome sequence inference methods in environments with varying biomass. Gibbons SM, editor. mSystems. 2019;4:15–9.

23. Prodan A, Tremaroli V, Brolin H, Zwinderman AH, Nieuwdorp M, Levin E. Comparing bioinformatic pipelines for microbial 16S rRNA amplicon sequencing. Seo J-S, editor. PLoS ONE. 2020;15:e0227434–19.

24. Martin M. Cutadapt removes adapter sequences from high-throughput sequencing reads. EMBnet j. 2011;17:10.

25. Murali A, Bhargava A, Wright ES. IDTAXA: a novel approach for accurate taxonomic classification of microbiome sequences. Microbiome. 3rd ed. BioMed Central; 2018;6:140–14.

26. Wright ES. Using DECIPHER v2.0 to analyze big biological sequence data in R. the R journal. 2016;8:352–9.

27. Bengtsson-Palme J, Ryberg M, Hartmann M, Branco S, Wang Z, Godhe A, et al. Improved software detection and extraction of ITS1 and ITS2 from ribosomal ITS sequences of fungi and other eukaryotes for analysis of environmental sequencing data. Bunce M, editor. Methods in Ecology and Evolution. 2013;4:914–9.

28. Sievers F, Higgins DG. Clustal Omega, accurate alignment of very large numbers of sequences. Methods Mol. Biol. 2014;1079:105–16.

29. Price MN, Dehal PS, Arkin AP. FastTree 2 - approximately maximum-likelihood trees for large alignments. PLoS ONE. 2010;5:e9490.

30. McMurdie PJ, Holmes S. phyloseq: an R package for reproducible interactive analysis and graphics of microbiome census data. PLoS ONE. 2013;8:e61217.

31. Oksanen J, Blanchet FG, Kindt R, Legendre P, Minchin PR, O'Hara RB, et al. vegan: Community Ecology Package. 2015.

32. Boers SA, Hays JP, Jansen R. Micelle PCR reduces chimera formation in 16S rRNA profiling of complex microbial DNA mixtures. Scientific Reports. 2015;5:14181.

33. Callahan BJ, Wong J, Heiner C, Oh S, Theriot CM, Gulati AS, et al. High-throughput amplicon sequencing of the full-length 16S rRNA gene with single-nucleotide resolution. Nucleic Acids Res. Oxford University Press; 2019;13:360–12.

34. Li C, Chng KR, Boey EJH, Ng AHQ, Wilm A, Nagarajan N. INC-Seq: accurate single molecule reads using nanopore sequencing. GigaScience. 2016;5:296–11.

35. Calus ST, Ijaz UZ, Pinto AJ. NanoAmpli-Seq: a workflow for amplicon sequencing for mixed microbial communities on the nanopore sequencing platform. GigaScience. 2018;7:1621–16.

36. D'Amore R, Ijaz UZ, Schirmer M, Kenny JG, Gregory R, Darby AC, et al. A comprehensive benchmarking study of protocols and sequencing platforms for 16S rRNA community profiling. BMC Genomics. BioMed Central; 2016;17:55.

37. Bakker MG. A fungal mock community control for amplicon sequencing experiments. Mol Ecol Resour. 2018;18:541–56.

38. Thiéry O, Vasar M, Jairus T, Davison J, Roux C, Kivistik P-A, et al. Sequence variation in nuclear ribosomal small subunit, internal transcribed spacer and large subunit regions of *Rhizophagus irregularis* and *Gigaspora margarita* is high and isolate-dependent. Mol Ecol. John Wiley & Sons, Ltd; 2016;25:2816–32.

39. Hurlbert SH. The nonconcept of species diversity: a critique and alternative parameters. Ecology. John Wiley & Sons, Ltd; 1971;52:577–86.

40. O'Hara RB. Species richness estimators: how many species can dance on the head of a pin? J Anim Ecology. 2005;74:375–86.

41. Chiu C-H, Wang Y-T, Walther BA, Chao A. An improved nonparametric lower bound of species richness via a modified good-turing frequency formula. Biom. 2014;70:671–82.

42. Edgar RC. UNBIAS: An attempt to correct abundance bias in 16S sequencing, with limited success. bioRxiv. Cold Spring Harbor Laboratory; 2017;10:57–23.

43. D'Amore R, Ijaz UZ, Schirmer M, Kenny JG, Gregory R, Darby AC, et al. A comprehensive benchmarking study of protocols and sequencing platforms for 16S rRNA community profiling. BMC Genomics. BioMed Central; 2016;17:55.

44. Quast C, Pruesse E, Yilmaz P, Gerken J, Schweer T, Yarza P, et al. The SILVA ribosomal RNA gene database project: improved data processing and web-based tools. Nucleic Acids Research. 2013;41:D590–6.

45. White TJ, Bruns T, Lee S, Taylor JL. Amplification and direct sequencing of fungal ribosomal RNA genes for phylogenetics. PCR protocols: a guide to methods and applications. 1990;18:315–22.

46. Ihrmark K, Bödeker I, Cruz-Martinez K, Friberg H, Kubartova A, Schenck J, et al. New primers to amplify the fungal ITS2 region–evaluation by 454-sequencing of artificial and natural communities. FEMS Microbiol. Ecol. 2012;82:666–77.

47. Cruaud P, Rasplus J-Y, Rodriguez LJ, Cruaud A. High-throughput sequencing of multiple amplicons for barcoding and integrative taxonomy. Scientific Reports. Nature Publishing Group; 2017;:1–12.

48. Kõljalg U, Nilsson RH, Abarenkov K, Tedersoo L, Taylor AFS, Bahram M, et al. Towards a unified paradigm for sequence-based identification of fungi. Mol Ecol. 2013;22:5271–7.

49. Community U. UNITE general FASTA release for Fungi.

Figure Legends

Figure 1: Overview of dadasnake workflow for paired-end Illumina sequencing of a fungal ITS region with inputs (configuration file, sample table and read files) and outputs (read numbers, graphical representations of quality and error models, rarefaction curves and "OTU tables", in biom, table and phyloseq format). The steps are configurable and alternative workflows exist, e.g. for single-end, non-Illumina datasets, or other target regions. Primer-removal and all post-DADA2-steps are optional. Colours represent the level of analysis: yellow – analysis per library/sample, bright green – analysis per run, sea green – analysis of the cummulated dataset; blue – analysis for the whole dataset with sample-wise documentation; note – the DADA2 block can be performed in pooled mode at the level of the whole dataset.

Figure 2: Comparison of mock-community composition with analysis results. a) Detection of prokaryotic genera at the highest sequencing depth; b) detection of fungal genera at the highest sequencing depth; c) number of detected prokaryotic ASVs plotted against the number of processed (non-chimeric) reads - black circles: ASVs of taxa from the mock community, grey circles: likely contaminant taxa; d) number of detected fungal ASVs against the number of processed (non-chimeric) reads; c & d) dotted lines indicate expected taxa richness.

Figure 3: Limitations of analysis. a & b) Misestimation of unobserved ASVs from final tables of reads per ASV per sample of the (a) prokaryotic without contaminants and (b) fungal mock communities analysed to different sequencing depths; c) missing correlation of real relative parts of the mock communities and detected relative abundances of prokaryotic genera; d) high coefficients of variation between relative abundances of taxa that are equally abundant in the fungal mock community.

Figure 1

Click here to download Figure Fig1.pdf

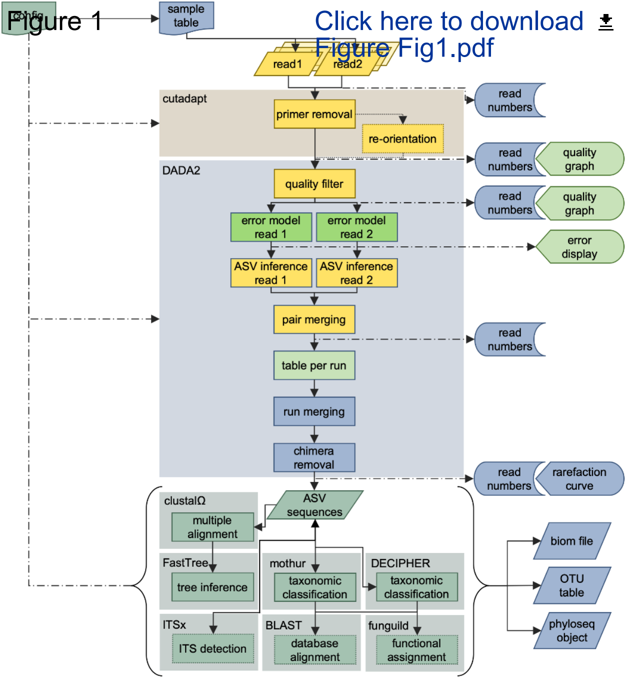

Figure 2

Click here to download Figure Fig2.pdf

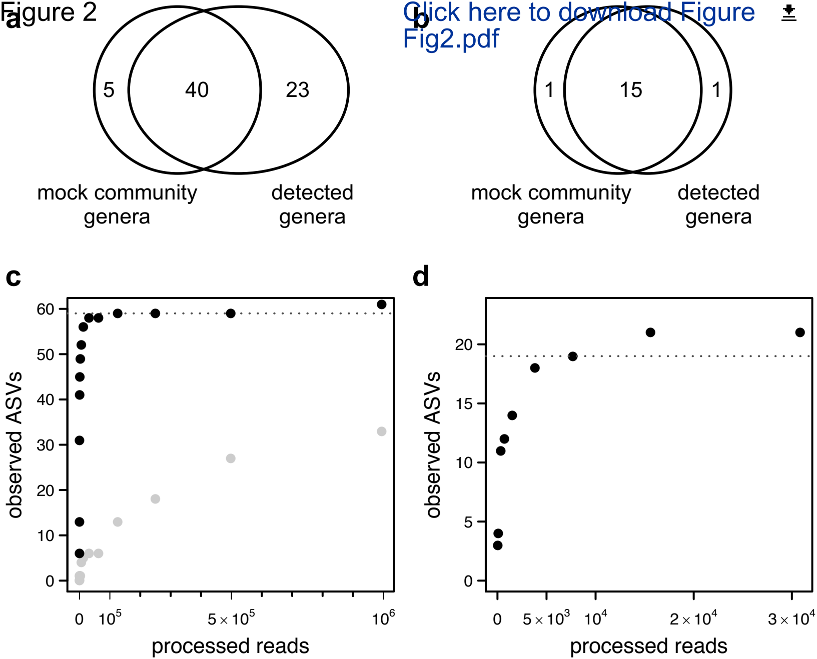

Figure 3

Click here to download Figure Fig3.pdf

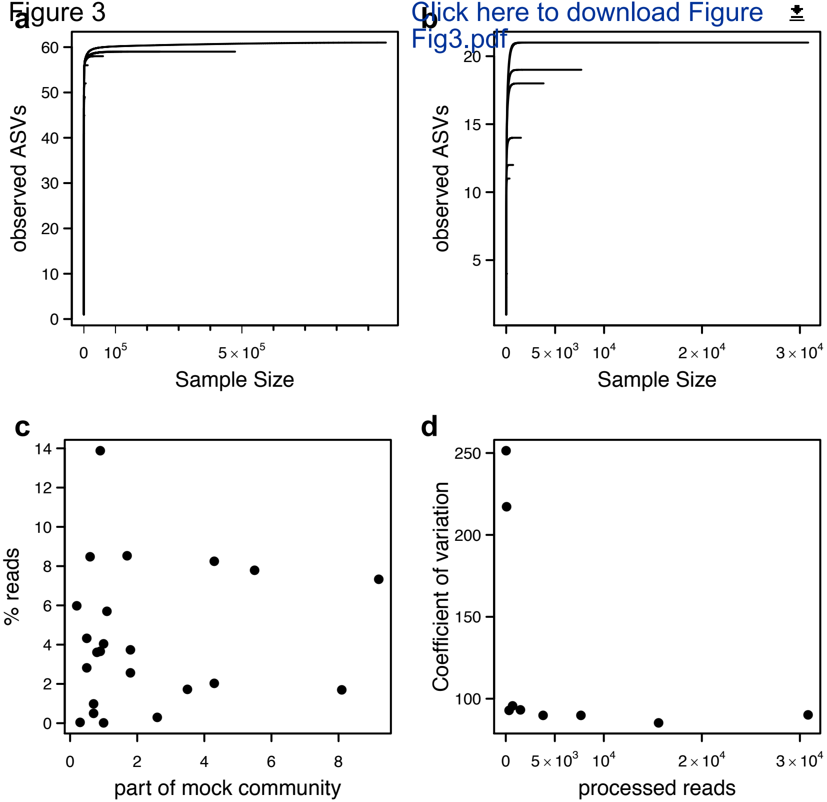

Supplementary Table 1

Click here to access/download
Supplementary Material
supplementary_table1-config.xlsx

Supplementary Table 2

Click here to access/download
Supplementary Material
supplementary_table2-groundTruthVsIdentification.xlsx
