# Supplementary material for: Dadasnake, a Snakemake implementation of DADA2 to process amplicon sequencing data for microbial ecology
Source: Gigascience. 2020 Nov 30;9(12):giaa135. doi: 10.1093/gigascience/giaa135 (PMC7702218; doi:10.1093/gigascience/giaa135)
Supplement: giaa135_GIGA-D-20-00147_Revision_1 [file giaa135_giga-d-20-00147_revision_1.pdf]

GigaScience

dadasnake, a Snakemake implementation of DADA2 to process amplicon sequencing data for microbial ecology

--Manuscript Draft--

**Manuscript Number:**	GIGA-D-20-00147R1
**Full Title:**	dadasnake, a Snakemake implementation of DADA2 to process amplicon sequencing data for microbial ecology
**Article Type:**	Technical Note
**Funding Information:**	Deutsche Forschungsgemeinschaft (FZT118) — Not applicable<br>Deutsche Forschungsgemeinschaft (BU 941/23-2) — Not applicable
**Abstract:**	Background: Amplicon sequencing of phylogenetic marker genes, e.g. 16S, 18S or ITS rRNA sequences, is still the most commonly used method to determine the composition of microbial communities. Microbial ecologists often have expert knowledge on their biological question and data analysis in general, and most research institutes have computational infrastructures to employ the bioinformatics command line tools and workflows for amplicon sequencing analysis, but requirements of bioinformatics skills often limit the efficient and up-to-date use of computational resources.<br>Results: dadasnake wraps pre-processing of sequencing reads, delineation of exact sequence variants using the favorably benchmarked, widely-used the DADA2 algorithm, taxonomic classification and post-processing of the resultant tables, and hand-off in standard formats, into a user-friendly, one-command Snakemake pipeline. The suitability of the provided default configurations is demonstrated using mock-community data from bacteria and archaea, as well as fungi.<br>Conclusions: By use of Snakemake, dadasnake makes efficient use of high-performance computing infrastructures. Easy user configuration guarantees flexibility of all steps, including the processing of data from multiple sequencing platforms. dadasnake facilitates easy installation via conda environments. dadasnake is available at https://github.com/a-h-b/dadasnake .
**Corresponding Author:**	Anna Heintz-Buschart<br>Helmholtz-Zentrum fur Umweltforschung UFZ<br>Halle, GERMANY
**Corresponding Author Secondary Information:**	
**Corresponding Author's Institution:**	Helmholtz-Zentrum fur Umweltforschung UFZ
**Corresponding Author's Secondary Institution:**	
**First Author:**	Christina Weißbecker
**First Author Secondary Information:**	
**Order of Authors:**	Christina Weißbecker<br>Beatrix Schnabel<br>Anna Heintz-Buschart
**Order of Authors Secondary Information:**	
**Response to Reviewers:**	Dear Hongling Zhou, dear GigaScience Editors,<br><br>Please find enclosed our revision of manuscript GIGA-D-20-00147, now entitled “dadasnake, a Snakemake implementation of DADA2 to process amplicon sequencing data for microbial ecology”, which we would like to re-submit for your consideration and potential publication as Technical Note in GigaScience.

Powered by Editorial Manager® and ProduXion Manager® from Aries Systems Corporation

We have now addressed all comments by the two reviewers, in particular by adding better descriptions of how our “dadasnake” workflow can be used, by discussing how it is distinguished from other workflows, and by including several additional case studies to evaluate its performance and demonstrate that our “dadasnake” workflow provides a flexible, single-command pipeline to facilitate the efficient analysis of large-scale amplicon sequencing or metabarcoding data.

We have attached a point-by-point response to the reviewers’ valuable comments to this letter. In light of the reviewers’ overall positive response and the improvements made according to their statements, we are hopeful that you will agree on the revised manuscripts’ interest for the readers of GigaScience and find it suitable for publication.

We have also submitted and revised the manuscript as a preprint on bioRxiv (BIORXIV/2020/095679).

Thank you in again for considering our manuscript, and we look forward to hearing from you.

Sincerely,

Anna Heintz-Buschart

Editor's and reviewers' comments below:

Your manuscript "Efficiently processing amplicon sequencing data for microbial ecology with dadasnake, DADA2 in Snakemake" (GIGA-D-20-00147) has been assessed by our reviewers. Both reviewers have requested a lot of additional work. In particular this work needs a better explanation and description on how to use it. As well as additional testing and comparisons to meet our criteria on computational work meeting state-of-the-art. We are giving you a chance to address these points, but if you need additional time we can close your file and await a resubmission later.

Reviewer reports:

Reviewer #1: General comments: In the manuscript, the authors presented a wrapper, dadasnake, for existing softwares to analyze amplicon data. I appreciate that dadasnake can facilitate the community analysis of the amplicon datasets. Also, I appreciate that the workflow was tested on a fungal mock community sequenced for this paper. However, I would like to address some concerns, mainly about the novelty of the study and benchmarking results. Other pipelines, both in snakemake (e.g. https://github.com/shu251/tagseq-qiime2-snakemake) or other platforms (i.e. https://doi.org/10.7287/peerj.preprints.27272v1), are available for the processing of amplicon datasets with dada2. Therefore, I would ask the authors to specify more about the novel aspect that dadasnake brings to the microbiome community, and to compare to existing pipelines. Regarding this, you may find my assessments on a number of aspects recommended by GigaScience reviewer guidelines. Also, at the end of the text I provided some minor specific comments.

Answer: We thank you for your appreciation of the dadasnake workflow and the recommendations for improving the manuscript. We have addressed all points in the manuscript as well as below, especially as regards the differences to the many other workflows for amplicon sequencing data analysis.
We would like to particularly thank you for pointing us to other amplicon sequencing analysis pipelines with in snakemake, which we would have liked to reference in the manuscript. While we don’t wish to discuss the details in the manuscript, we’d like to point out that we could not run the tagseq-qiime2-snake pipeline due to undocumented dependencies.

1.Is the rationale for collecting and analyzing the data well defined?

The work describes fairly well the previous work on the field. I appreciate that the authors sequence a house-built fungal community for testing their workflow.

Powered by Editorial Manager® and ProduXion Manager® from Aries Systems Corporation

Answer: We are very sorry, but we have not built the fungal community ourselves. The source of the fungal mock-community was clearly stated in the manuscript, and we have pointed to this again in the methods section to avoid the impression that we did build the community (L 232).

2.Is it clear how data was collected and curated?

Some details about composing the fungal mock community are missing (e.g. Which fungi are there, what are the percentage abundances of each fungi and how did you count and merge fungal cells or DNA?).

Answer: As explained above, the mock community was obtained from Matt Bakker, who has detailed this in the cited publication (ref 42 in revised reference counting). We had previously mentioned the fact that it is even, i.e. the same amount of each fungal species should be present, in the results section (L 203) and now mention it additionally in the methods section (L 232). The list of species in both analysed mock-communities had already been included in the supplementary materials, and we now point to this in the results section (L 162, 178) and methods section (L 217, 233).

Descriptions of some analysis are also missing (e.g. coefficient of variation). Otherwise, the workflow is explained well.

Answer: Thank you, also for pointing out this omission, we've added the equation in L 299-300.

3.Is it clear - and was a statement provided - on how data and analyses tools used in the study can be accessed?
All the scripts are available in the github page of dadasnake. Also, accession numbers for the data are provided.

Answer: Thank you.

4.Are accession numbers given or links provided for data that, as a standard, should be submitted to a community approved public repository?

Accession numbers are provided for the data.

Answer: Thank you.

5.Is the data and software available in the public domain under a Creative Commons license?

The code is stored in a github repository with a GNU general public license.

Answer: Thank you.

6.Are the data sound and well controlled?

The details about the archaea part of the mock community is missing.

Answer: We are very sorry for lumping archaea and bacteria together – we do know better. We have now reported them separately (L 161, 164, 217).

7.Is the interpretation (Analysis and Discussion) well balanced and supported by the data?

Overall, current literature, results and limitations of the study interpreted and discussed well. The authors also provided references wherever necessary.

Answer: Thank you for this appraisal.

However, benchmarking the accuracy of the wrapper might be unnecessary (Figure2-3) since the wrapper relies on softwares and packages whose accuracy is already tested (e.g. dada2, ITSx). In my opinion, the main point of the wrapper here is not to

Powered by Editorial Manager® and ProduXion Manager® from Aries Systems Corporation

	increase the accuracy but to facilitate the usage of the amplicon analysis pipelines. Therefore, it would be more interesting to compare dadasnake to vanilla dada2 (or other dada2 implementations e.g. in QIIME2), and to compare dadasnake to other OTU pipelines such as QIIME2, mothur, LotuS etc.<br><br>Answer: Thank you for this suggestion. We agree that the accuracy of DADA2 has already been evaluated several times, which is why we're using it in dadasnake – this is also the same whether wrapped in QIIME2 or in vanilla R. We feel that it is nevertheless important to demonstrate that the pipeline as a whole produces realistic results and provide ideas on the limitations of the process in the manuscript. Regarding ease of use, this is difficult to assess objectively and depends strongly on the background of the users. However, according to your comment, we have now included a discussion of the points where dadasnake is easier to use than other pipelines and where it is more flexible (L 63-65, 94-120).<br><br>One main component is missing: the computational time, memory usage, hdd usage is not discussed at all and could be used instead of the current Fig. 3 to illustrate the efficiency of the pipeline.<br><br>Answer: We have now added examples for three differently sized datasets and report the resource use in a new paragraph and figure (L 125-156, Figure 2).<br><br>8.Are the methods appropriate, well described, and include sufficient details and supporting information to allow others to evaluate and replicate the work?<br><br>Methods for the workflow are well described. Also, in the github page, parameters are well-explained and possible user errors are stated. However, some points in the methods and results sections deserve more attention which we explained at 7 and 9.<br><br>Answer: Thank you. Please see our responses in 1,2,6,7,9 for those points.<br><br>9.What are the strengths and weaknesses of the methods?<br>Bacterial and archaean mock community data set:<br>What happened to the archeal reads? I did not see that it is reported further in the results, this should be further discussed.<br><br>Answer: We have revised this, as mentioned in point 6 above.<br><br>Fungal mock community sequencing:<br>Was ITSx also used in the analysis? It was mentioned in the implementation section; however, it was not mentioned in the analysis of the fungal mock community and how it performed/helped. Also, could you please provide further details on composing of the mock community as we mentioned at point 2?<br><br>Answer: Indeed, we used ITSx in the analysis of the fungal mock community. In the case of the mock community, it doesn't have an effect on the final result. It is however useful when the real dataset contains non-fungal ASVs, which is often the case in real samples, e.g. from soil. We've now added this detail (L 248).<br><br>10.Have the authors followed best-practices in reporting standards?<br>The methods used in the dadasnake workflow are amenable. Also, the authors provided the conda environment for dadasnake, which facilitates easy installation.<br><br>Answer: Thank you.<br><br>11.Can the writing, organization, tables and figures be improved?<br><br>The quality of the written English is amenable and the manuscript is organized in a logical way. However, figures may be made more comprehensive:<br>Figure2: Why ASVs from the fungal mock community are not denoted in Figure 2d?<br><br>Answer: Figure 3d (formerly figure 2d) does show the numbers of fungal ASVs detected and expected from the fungal mock community. The legend of this figure has been clarified now.

Also, bacterial false positives are too many, the results in the dada2 paper looked a lot better and this might be a very interesting discussion to add to the paper. How to be sure that this is mainly due to contamination? Please consider rephrasing "likely contaminants" - what is the evidence for this? Please discuss this in the manuscript.

Answer: We were also originally surprised by the number of false positives, especially compared to earlier benchmarks. We've checked the distance between the potential contaminant ASVs and the nearest ASVs from expected taxa and found them to be too unrelated to be down to sequencing errors. We are confident that these are not artificial ASVs but represent contaminating DNA. As we've previously stated, this has been observed before. We now discuss in some more detail (L 165-169) and have added a column in the results in supplementary table 3 to indicate previously established contaminants.
Furthermore, the ASVs are classified to genus or species level, which we think would be highly unlikely if they were artificial ASVs – we don't have a reference or analysis to back this specific claim up, so we are not discussing the exact classification in the manuscript.

Overall, Figure 2 could be clearer, please consider adding a legend etc.

Answer: Thank you for the comment. We've reworked this figure when we merged it with figure 3 (now figure 3).

Figure 3: I am not sure whether Figure3 A-B adds further value to the manuscript, considering Figure 2C-D.

Answer: We've combined Figure 3c/d with Figure 2 (now Figure 3) and removed Figure 3a/b. Instead, we point to the DADA2 documentation, which mentions this effect, too (L 193).

Figure 3c) Please explain better what is x- axis: "part" of mock community.

Answer: Thank you for pointing out that this is unclear, we've changed the axis label to read "percentage of mock-community" now (Fig 3 e).

Figure 3D) How did you calculate a coefficient of variation? Why not make Figure 3C in the same way?

Answer: The reason to use the coefficient of variation is that we expect equal amounts for the fungal mock community. Compared to Fig. 3E, we'd have the same value along the x-axis for all taxa. We've made this more clear in the text now (L 201-204).

12.When revisions are requested.

Minor revision is requested.

Answer: Thank you.

13.Are there any ethical or competing interests issues you would like to raise?

Not detected.

Specific Comments:
Full title: Why efficient? The efficiency of the pipeline is not really tested in this study. This should be benchmarked against other pipelines (even those not using dada2 as clustering), and against vanilla dada2.

Answer: Thank you for this suggestion. We've adjusted the title accordingly.

Abstract: line2-3: what do you mean by "structure of microbial communities". Please use a less ambiguous term.

Answer: Thank you for pointing out that this term that it well established in microbial

Powered by Editorial Manager® and ProduXion Manager® from Aries Systems Corporation

ecology is not informative to most readers of GigaScience. We've translated it to standard English now.

Background:
L7: lack of quantitative value → lack of absolute abundances

Answer: Thank you for this suggestion. We've adjusted the sentence accordingly (L 45).

L8: an overview of microbial diversity and composition

Answer: Thank you for this suggestion. We've adjusted the sentence accordingly (L 46).

"A second limitation is that relative abundances of ASVs are not reflective of the actual abundance of the sequenced taxa, which varied for the prokaryotic mock community and were equal in the fungal mock community." → this is rather a limitation of the amplicon sequencing.

Answer: Thank you for this suggestion. We've adjusted the sentence accordingly (L 199).

Reviewer #2: "Dadasnake" is a snakemake workflow that implements the DADA2 method for processing amplicon sequencing data, as well as additional tools for assigning taxonomy, creating phylogenetic trees, and inferring function from denoised tables of common taxonomic barcode genes (e.g. 16S, ITS). The benefits of the dadasnake workflow are in its combination of snakemake, which efficiently and reproducibly makes use of high-performance computing (HPC) resources, with widely used and well-validated amplicon sequencing tools that would otherwise be difficult for many to easily and effectively use in an HPC environment. The goal of this project is laudable. The documentation around some of the core tools incorporated here, in particular DADA2, are mostly centered around operation on a personal computer, and for non-experts the deployment of these tools on HPCs can be a bridge too far.

Answer: Thank you for your acknowledgement of dadasnake's purpose.

However, I think the current manuscript suffers from a critical weakness in that the dadasnake workflow itself is not sufficiently explained and explored, particularly when it comes to the ways in which potential (non-expert) users might interact with it while configuring parameters, assessing whether processing happened as expected, and diagnosing problems when they arise. There is significantly more information on these critical topics in the online documentation available at https://github.com/a-h-b/dadasnake, which is fine for the details. But… I think the paper itself needs a clear description of parameterization including the yaml file format that would allow a non-expert to understand how they might use that format to change relevant parameters, a fuller description of the intermediate outputs/visualizations that snakemake or tools like DADA2 create within the dada2snake workflow that would allow a non-expert to understand how they might go about fixing poorly chosen parameters or diagnosing the step at which the workflow failed, and what they could do once something has gone wrong.

Answer: Thank you for this suggestion. We've originally wanted to keep the manuscript short. We now realize that we have to bridge between the short description and the documentation in the github repository. We have now expanded the section on configuring and running dadasnake (L 95-101, 111-123), including more explicit references to the supplementary material and additional explanatory supplementary tables. More detailed information will still best be accessed via the github page, which has the added advantage of us being able to update the information according to users' comments.

Powered by Editorial Manager® and ProduXion Manager® from Aries Systems Corporation

Furthermore, some evaluation of the ability of dadasnake to efficiently use HPC hardware would be another very nice element. Simple question: How much does wall-time decrease when using dadasnake on 4 nodes vs. 40 nodes? More complex question: Are there particular steps that become rate limiting in very high resource environments (maybe tree-ing?).

Answer: Thank you for this suggestion. We have added information on the resource use of dadasnake (L 125-155, 250-289).

The paragraph above is my biggest concern. Other itemized issues/suggestions below, with *(s) appending those that are more important.

** What is the plan for the maintenance or continued development of the conda environment behind dadasnake? Is it currently pinned to defined versions of each package? Or will it update along with the packages as they appear on bioconda? How will testing of package updates be done?

Answer: Thank you for this suggestion. The workflow is indeed pinned to fixed versions of each package. Since we are heavily using the workflow, it's in our own interest to update and test when new versions, in particular of DADA2 become available. For example, the latest version of dadasnake has moved to R4.0.2. We have added our future plans in L 87-89.

* What is the user support plan when folks have problems with the workflow? The documentation at the website is acceptable, but I guarantee there will be problems folks have they won't be able to solve via the documentation. What then?

Answer: Since the original submission, we have expanded the documentation in the github page, including trouble-shooting. We've also attended to issues posted there.

* Default parameter choices become the parameters used by the vast majority of any workflow. How were the defaults for dadasnake chosen? I can see at least one default parameter (truncQ=13) that is not the default suggested by the tool itself.

Answer: Thank you for pointing out this difference. According to your comment, we've changed the DADA2 default settings within dadasnake to the DADA2 defaults (in particular in the filtering step, where they differed from the DADA2 defaults).
We've chosen the settings in the example config files based on the accuracy of the results in the reported (and other) mock datasets. We've kept these changed parameters in the example configurations, as they work best in our hands.

"Pooled analysis can alternatively be chosen in dadasnake and is recommended for more error prone technologies such as 454 or third generation long reads.": Is this the authors recommendation? If so say "we recommend". If not, can the authors provide a source or citation for this recommendation?

Answer: We've edited the sentence according to your suggestion (L 104-106). It is our observation that non-pooled analysis of error-prone reads leads to different ASVs in each sample. We actually provide a mock community dataset from 454 sequencing in the supplementary material (now referenced in L 108). However, we don't want to inflate the manuscript with information that will not be of interest to most users, so we've not discussed it further.

"Exact sequencing variants" -> "exact sequence variants", several places.

Answer: Oh dear, we're sorry and have corrected this throughout.

Snakemake is central to this whole thing. Perhaps it should be a keyword, and perhaps should be described a bit more in the "Implementation" section.

Answer: We've expanded the Snakemake description in the usage paragraph (L 84-91, 118-121). Snakemake is part of the title, so it'll be visible for searches and bibliographical applications.

Powered by Editorial Manager® and ProduXion Manager® from Aries Systems Corporation

	Use case/Limitations: As I mentioned above, this is narrowly focused on how DADA2/amplicon sequencing did at measuring a mock community, and this tells us little about how dadasnake performs, other than it can successfully run DADA2 on a single test dataset.<br><br>Answer: According to your and the other reviewer's comment, we've added useful information on the different scales of datasets dadasnake can handle (L 125-156, 250-289).
**Additional Information:**	
**Question**	**Response**
Are you submitting this manuscript to a special series or article collection?	No
**Experimental design and statistics**<br><br>Full details of the experimental design and statistical methods used should be given in the Methods section, as detailed in our Minimum Standards Reporting Checklist. Information essential to interpreting the data presented should be made available in the figure legends.<br><br>Have you included all the information requested in your manuscript?	Yes
**Resources**<br><br>A description of all resources used, including antibodies, cell lines, animals and software tools, with enough information to allow them to be uniquely identified, should be included in the Methods section. Authors are strongly encouraged to cite Research Resource Identifiers (RRIDs) for antibodies, model organisms and tools, where possible.<br><br>Have you included the information requested as detailed in our Minimum Standards Reporting Checklist?	Yes
**Availability of data and materials**<br><br>All datasets and code on which the conclusions of the paper rely must be	Yes

either included in your submission or deposited in publicly available repositories (where available and ethically appropriate), referencing such data using a unique identifier in the references and in the "Availability of Data and Materials" section of your manuscript.<br><br>Have you have met the above requirement as detailed in our Minimum Standards Reporting Checklist?	

Powered by Editorial Manager® and ProduXion Manager® from Aries Systems Corporation

Manuscript

Click here to access/download;Manuscript;201104_draft_noFig_lineNumber

dadasnake, a Snakemake implementation of DADA2 to process amplicon sequencing data for microbial ecology

Christina Weißbecker[1], *Beatrix Schnabel*[1], *Anna Heintz-Buschart*[2,1]*

*corresponding author, email: anna.heintz-buschart@ufz.de; +49-345-558-5225

[1]Helmholtz Centre for Environmental Research GmbH - UFZ, Department of Soil Ecology

[2]German Centre for Integrative Biodiversity Research (iDiv) Halle-Jena-Leipzig, Bioinformatics Unit

ORCIDs:

Christina Weißbecker: 0000-0002-8212-6170

Anna Heintz-Buschart: 0000-0002-9780-1933

Abstract

Background: Amplicon sequencing of phylogenetic marker genes, e.g. 16S, 18S or ITS rRNA sequences, is still the most commonly used method to determine the composition of microbial communities. Microbial ecologists often have expert knowledge on their biological question and data analysis in general, and most research institutes have computational infrastructures to employ the bioinformatics command line tools and workflows for amplicon sequencing analysis, but requirements of bioinformatics skills often limit the efficient and up-to-date use of computational resources.
Results: dadasnake is a user-friendly, one-command Snakemake pipeline that wraps the pre-processing of sequencing reads and the delineation of exact sequence variants by using the favorably benchmarked and widely-used the DADA2 algorithm with a taxonomic classification and the post-processing of the resultant tables, including hand-off in standard formats. The suitability of the provided default configurations is demonstrated using mock-community data from bacteria and archaea, as well as fungi.
Conclusions: By use of Snakemake, dadasnake makes efficient use of high-performance computing infrastructures. Easy user configuration guarantees flexibility of all steps, including the processing of data from multiple sequencing platforms. dadasnake facilitates easy installation via conda environments. dadasnake is available at https://github.com/a-h-b/dadasnake .

Keywords
rRNA gene sequence analysis; denoising; exact sequence variants; R; pipeline; microbiome; community structure

Findings

Background

Since the first reports 15 years ago [1], high-throughput amplicon sequencing has become the most common approach to monitor microbial diversity in environmental samples. Sequencing preparation, throughput and precision have been consistently improved, while costs have decreased. Computational methods have been refined in the recent years, especially with the shift to exact sequence variants and better use of sequence quality data [2,3]. While amplicon sequencing can have severe limitations, such as limited and uneven taxonomic resolution [4,5], over- and underestimation of diversity [6,7], lack of absolute abundances [8,9] and missing functional information, amplicon sequencing is still considered the method of choice to gain an overview of microbial diversity and composition in a large number of samples [10,11]. Consequently, the sizes of typical amplicon sequencing datasets have grown. In addition, synthesis efforts are undertaken, requiring efficient processing pipelines for amplicon sequencing data [12]. Due to the unique, microbiome-specific characteristics of each dataset and the need to integrate the community structure data with other data types, such as abiotic or biotic parameters, users of data processing tools need to have expert knowledge on their biological question and statistics. It is therefore desirable that workflows should be as user-friendly as possible. Several widely used tool collections e.g. QIIME 2 [13], mothur [14], usearch [15], vsearch [16], and one-stop pipelines, e.g. lOTUs [17], with new approaches continually being developed, e.g. OCToPUS [18], PEMA [19]. Typically, workflows balance learning curves, configurability and efficiency.

Purpose of dadasnake

dadasnake is a workflow for amplicon sequencing data processing into annotated exact sequence variants. It is set up with microbial ecologists in mind, to be run on high-performance clusters without the users needing any expert knowledge on their operation. dadasnake is implemented in Snakemake [20] using the conda package management system. Consequently, it features a simple installation process, a one-command execution, and high configurability of all steps with sensible defaults. dadasnake includes example workflows for common applications and produces a unique set of useful outputs, comprising relative abundance tables with taxonomic and other annotations in multiple formats, reports on the data processing and visualizations of data quality at each step. The workflow is open-source, based on validated, favourably benchmarked tools.

Implementation

The central processing within dadasnake wraps the DADA2 R package [21], which accurately determines sequence variants [22-24]. The dadasnake wrapper eases DADA2 use and deployment on computing clusters without the overhead of larger pipelines with DADA2 such as QIIME 2 [13]. Within dadasnake, the steps of quality filtering and trimming, error estimation, inference of sequence variants, and, optionally, chimera removal are performed (Figure 1). Prior to quality filtering, dadasnake optionally removes primers and re-orients reads using cutadapt [25]. Taxonomic classification is realized using the reliable naïve Bayes classifier as implemented in mothur [14], or by DECIPHER [26,27] with optional species-identification in DADA2. BLAST [28] can optionally be used to annotate all or only unclassified sequence variants. The sequence variants can be filtered based on length, taxonomic classification or recognizable regions, namely ITSx [29] before downstream analysis. For downstream analyses, a multiple alignment [30] and FastTree-generated tree [31] can be integrated into a phyloseq [32] object. Alternatively, tab-separated or R tables and standardized BIOM format (https://biom-format.org/index.html) are generated. dadasnake records statistics, including numbers of reads passing each step, quality summaries, error models, and rarefaction curves [33]. All intermediate steps and configuration settings are saved for reproducibility.
Reproducibility, user-friendliness, and modular design are facilitated by the Snakemake framework, a popular workflow manager for reproducible and scalable data analyses Snakemake (RRID:SCR_003475)[20]. Snakemake also generates html reports, which store code, version numbers, the workflow and links to results. DADA2 and the other tools are packaged in conda environments to facilitate installation. For reasons of reproducibility, dadasnake uses fixed versions of all tools, which are regularly tested on mock-datasets and updated when improvements become available. Snakemake also ensures flexible use as single-threaded local workflow or efficient deployment on a batch scheduling system. Currently slurm and univa/sun grid engine scheduler configurations are defined for dadasnake.

dadasnake configuration and execution

The whole dadasnake workflow is started with a single command ("dadasnake -c configuration.yaml"). The user provides a tab-separated table with sample names and input files, as well as a configuration file in the simple, human-readable and -writable YAML format (see Supplementary file 1 for a worked example) to determine which steps should be taken and with what settings (a description of all configurable parameters in Supplementary table 1). dadasnake is highly configurable compared to other Snakemake-based amplicon sequencing workflows, e.g. Hundo [34]. To facilitate its use, dadasnake provides easily adjustable, tested default settings and configuration files for several use cases.

dadasnake can use single end or paired end data. DADA2 can be efficiently employed by parallelizing most steps by processing samples individually (https://benjjneb.github.io/dada2/bigdata.html). Pooled analysis can alternatively be chosen in dadasnake and we recommend it for more error prone technologies such as 454 or third generation long reads. While DADA2 has been designed for Illumina technology [21], dadasnake has been tested on Roche pyrosequencing data [35] and circular consensus Pacbio [36] and Oxford Nanopore data [37,38] (see supporting material). dadasnake provides example configurations for these technologies and for Illumina-based analysis of 16S, ITS and 18S regions of bacterial and fungal communities.

dadasnake offers a range of different output formats for easy integration with downstream analysis tools. Tab-separated or R tables and standardized BIOM format [39], or a phyloseq [32] object are generated as final outputs in the user-defined output directory (description of all outputs in Supplementary table 2). Visualizations of the input read quality, read quality after filtering, the DADA2 error models and rarefaction curves of the final dataset are also saved into a stats folder within the output. The numbers of reads passing each step are recorded for trouble shooting. All intermediate steps and configuration settings are saved for reproducibility and to restart the workflow in case of problematic settings or datasets, so hard disk requirements are about 1.3-fold the input data. The Snakemake-generated html report contains all software versions and settings to facilitate the publication of the workflow's results (see supporting material).

Snakemake provides detailed error reports and the logs of each step are recorded during runs. E-mail notifications of start and finishing can be sent. Users can find trouble shooting help and file issues [40].

Use cases: performance

To demonstrate dadasnake's performance, public datasets of different scales were processed. The performance of dadasnake depends strongly on the number of reads, number of samples, number of ASVs, and the required processing steps.

Small datasets can be run on single cores with less than 8 GB RAM, but profit from dadasnake's parallelization. For example, a 24-sample dataset with 2.9 million 16S rRNA V4 reads [41] could be completely processed, including preprocessing, quality filtering, ASV determination, taxonomic assignment, treeing, visualization of quality, and hand-off in various formats with a total walltime of 150 minutes. Running time was reduced to 100 minutes, when four cores were used, especially due to the parallelization of the preprocessing and ASV determination steps (Fig. 2 a&b). Hardware requirements for small datasets are minimal, including small personal laptops. A medium-sized ITS1 dataset (267 samples with a total of 46.8 million reads [42]) could be processed in just under 4 hours on four 8 GB cores, including quality filtering, ASV determination, extraction of ITS1, taxonomic assignment, visualization of quality, and hand-off in various formats (Fig. 2 c). While the system walltime was similar, the use of 15 cores reduced the runtime by a factor 2 (Fig. 2 d).

Generally speaking, dadasnake's parallelization of primer trimming, quality filtering, and ASV determination leads to shortened running times, while some steps, like merging of the ASV results of the single samples and all processing of assembled ASV tables, such as chimera removal, taxonomic annotation and treeing, are run sequentially. While dadasnake requests more cores for steps that use parallelized tools, such as ITSx or treeing, the speed-up is usually incremental. Of note for users of shared cluster environments, dadasnake does not occupy cores idly, e.g. when only a single core is used for merging of runs and chimera removal (Fig. 2 b-d) the other cores are available to other users, leading to high overall efficiency (> 90%).

dadasnake is able to preprocess reads, report quality, determine ASVs, and assign taxonomy for very large datasets, e.g. the original 2.1 billion reads in >27,000 samples of the Earth Microbiome Project publication [12] within 87 real hours on only up to 50 CPU cores. Due to the independent handling of the preprocessing, filtering and ASV definition steps, the number of input samples only prolongs the run time linearly. Sample merging and handling of the final table, however, requires more RAM the more unique ASVs and samples are found (e.g. > 190 GB for the >700,000 ASVs in the >27,000 samples of the Earth Microbiome Project). Tree building was not possible for this dataset on our infrastructure. For very large datasets it is therefore advisable to filter the final table before postprocessing steps.

Use cases: accuracy

To demonstrate dadasnake's potential to accurately determine community composition and richness, two mock community datasets from Illumina sequencing of bacterial and archaean [43] and fungal [44] DNA were analysed (compositions displayed in Supplementary table 3). In both cases, the genus-level composition was determined mostly correctly (Figure 2 a&b; Supplementary table 3). One fungal taxon and two archaeal and three bacterial taxa were not detected at all, likely because they were not amplified. False positive bacterial genera were unrelated to the taxa in the mock-community and contained several human/skin-associated taxa, like *Corynebacterium* and *Staphylococcus*, as well as commonly detected sequencing contaminants like Rhizobiaceae and *Sphingomonas* (see overlap with [45] in Supplementary table 3). The large number of false-positives was therefore likely caused by contaminations in the bacterial dataset which have been observed in this dataset before [24]. For the fungal dataset, one *Fusarium* sequence was misclassified as *Giberella.* In the same settings, the ASV richness was inferred close to correctly at 59 and 19 prokaryotic and fungal ASVs, respectively (ignoring the contaminants; Figure 2 c&d).

Next to accurate information on taxonomic composition and taxon richness, recognition of closely related strains is required from amplicon sequence processing tools. Six bacterial genera were represented by two strains each in the bacterial dataset and recognized as such by ASVs. In the case of three prokaryotic genera, the true diversity was not resolved by ASVs, with three *Thermotoga* strains and two *Salinispora* and two *Sulfitobacter* strains conflated as two and one strains, respectively (Supplementary table 3). Micro-diversity was correctly identified for two strains of *Aspergillus* and the three *Fusarium strains* (although one was misclassified) for the fungal dataset. Strain-diversity was overestimated for the fungal dataset in *Rhizophagus irregularis,* which is known to contain within-genome diversity of ribosomal RNA gene sequences [46]. Overall, dadasnake returns accurate results for taxonomic composition, richness and micro-scale diversity within the limits of taxonomic resolution within short regions.

Use cases: limitations

The analysis of the mock community data also revealed limitations of the approach in general. A commonly used approach to detect underestimation of richness at low sequencing depths is to plot rarefaction curves or use richness estimators [47-49], which use subsamples of the assigned reads to model how much the addition of further sequencing would increase the observed richness. However, the statistical requirements for delineation of ASVs mean that not all sequenced taxa are represented by an ASV in a given data set [50]. This in turn leads to the flattening of rarefaction curves derived from finished ASV tables, although an increase in real sequencing depth would lead to a greater number of observed ASVs (Figure 3 c&d). Richness estimates and rarefaction curves based on DADA2 datasets need to be handled with caution and whenever richness estimates are essential should be based on subsamples that are processed by DADA2 independently rather than post-hoc models.

A second limitation, common to amplicon sequencing, is that relative abundances of ASVs are not reflective of the actual abundance of the sequenced taxa, which varied for the prokaryotic mock community and were equal in the fungal mock community. Specifically, the relative abundance of the prokaryotic taxa did not correlate with the relative abundance of reads (Figure 2 e). The relative abundance of reads for the fungal taxa varied by several orders of magnitude, despite equal inputs

(Figure 3 f). There are numerous reasons for misrepresentation of abundances by PCR-based analyses [51]. Of note, the variation in the relative abundance estimates is observed to be highest at low sequencing depths (Figure 3 e&f). Therefore, whenever comparisons of relative abundances within samples are undertaken, it is necessary to, at the least, ensure that sequencing depths of all samples are sufficient to reach stable estimates. However, the analysis of the mock community case studies also suggests that true relative abundances can never be determined, which should be accounted for in experimental design and interpretation.

Methods

Bacterial and archaean mock community dataset

The largest library of the Illumina sequencing datasets of a 59 species mock-community [52], comprising 10 archaea and 49 bacteria (composition see Supplementary table 3) was retrieved from the European Nucleotide Archive ENA under accession ERR777696. The ground-truth composition of the mock-community was manually extracted from the publication and the taxonomic names adapted to the convention of the SILVA v. 138 database [53]. To analyse the effect of sequencing depth on the recovery of the mock-community, the dataset was subsampled to 100, 200, 500, 1,000, 2,000, 5,000, 10,000, 20,000, 50,000, 100,000, 200,000, 400,000, 800,000, and 1,600,000 read pairs.
The same configuration was used to run dadasnake on all subsamples. The most important settings include removal of the primers from either read (515F, specified as 5-GTGYCAGCMGCCGCGGTAA, and 806R, specified as 5-GGACTACNVGGGTWTCTAAT, with a maximum of 20 % mismatch); truncation of the reads at positions with a quality below 13, before removal of forward and reverse reads with less than 170 and 130 nt length, respectively, and truncation to these lengths before removal of reads with an expected error above 0.2; a minimum of 12 bp overlap was required for merging of denoised sequences; chimeras were removed on consensus.

Fungal mock community sequencing

The ITS2 region of an even 19 species fungal mock community [44] provided by Matt Bakker (composition see Supplementary table 3), was amplified using the primers F-ITS4 5-TCCTCCGCTTATTGATATGC [54] and R-fITS7 5-GTGARTCATCGAATCTTTG [55] modified with heterogeneity spacers according to [56]. Amplicon libraries were prepared using the Nextera XT kit (Illumina) and sequenced on an Illumina MiSeq (Illumina MiSeq System, RRID:SCR_016379) with v.3 chemistry at 2 x 300 bp. Sequencing was performed in triplicates and all reads were pooled for the analysis presented here. The sequencing data is accessible at the NCBI Short Read Archive under BioProject accession PRJNA626434. The ground-truth composition of the data was manually extracted from the publication and the taxonomic names were adjusted to the ones used in the Unite 8.0 database. To analyse the effect of sequencing depth on the recovers of the mock-community, the dataset was subsampled to 100, 200, 500, 1,000, 2,000, 5,000, 10,000, 20,000 and 40,000 reads.
The same configuration was used for running dadasnake on all subsamples. The most important settings were: removal of the primers from either read with a maximum of 20 % mismatch; truncation of the reads at positions with a quality below 15, before removal of reads with less than 70 nt length and removal of reads with an expected error above 3; a minimum of 20 bp overlap was required for merging of denoised sequences; chimeras were removed on consensus; ITSx was run on the ASVs which would remove non-fungal ASVs (which did not occur in the mock-community).

Performance testing

To demonstrate dadasnake's performance on a small laptop computer, a small data set of 24 16S rRNA gene amplicon sequences from a local soil fertilization study [41] were downloaded from the NCBI short read archive (PRJNA517390) using the fastq-dump function of the SRA-toolkit. Using the settings optimized for the bacterial mock-community, dadasnake was run either on a computer cluster using up to 1 or 4 threads with 8 GB RAM each, or without cluster-mode on three cores of a laptop with an Intel i5-2520M CPU with 2.5 GHz and 8 GB shared RAM.
To compare performance of dadasnake on a medium sized study in different settings, ITS1 amplicon sequences of 267 samples measured using Illumina HiSeq technology in a global study on fertilization effects [42] were downloaded from the NCBI short read archive (PRJNA272747) using the fastq-

dump function of the SRA-toolkit. Owing to the variable length of the ITS1 region, reads were not truncated to a specified length, but trimmed to a minimum per-base quality of 15 (also discarding reads with a higher maximum expected error than 3). After error modelling and ASV construction per sample, read pairs were merged with at least 20 bp overlap, allowing for 2 mismatches. After table set-up, the ITSx classifier was run to remove non-fungal ASVs before taxonomic annotation (using the mothur [14] classifier; configuration see Supplementary file 1). The same runs were performed on either a compute cluster using up to 50 threads or only up to 4 threads with 8GB RAM each.
27,081 samples analysed by the Earth Microbiome Project [12] stored under accessions ERP021896, ERP020023, ERP020508, ERP017166, ERP020507, ERP017221, ERP016412, ERP020884, ERP020022, ERP020510, ERP017438, ERP016395, ERP020539, ERP016468, ERP020590, ERP020021, ERP020587, ERP020560, ERP020589, ERP017176, ERP017220, ERP017174, ERP016405, ERP020591, ERP021691, ERP016416, ERP022167, ERP021699, ERP016495, ERP022245, ERP016748, ERP016749, ERP016752, ERP016540, ERP006348, ERP016543, ERP016746, ERP016586, ERP016735, ERP021864, ERP016588, ERP016587, ERP016539, ERP016734, ERP016492, ERP003782, ERP016607, ERP016581, ERP016557, ERP016464, ERP016542, ERP016541, ERP016591, ERP016854, ERP016852, ERP016286, ERP016451, ERP023684, ERP016869, ERP010098, ERP016879, ERP016883, ERP016466, ERP016496, ERP016880, ERP016455, ERP016900, ERP016924, ERP016923, ERP016925, ERP016927, ERP016469, ERP016329, ERP016926, ERP021540, ERP021541, ERP021542, ERP021543, ERP021544, ERP021545, ERP016937, ERP016131, ERP016483, ERP016252, ERP022166, ERP016414, ERP016472, ERP023686, ERP017459, ERP016287, ERP016285, ERP005806, ERP021895, ERP016384, ERP016491, and ERP006348 were downloaded from the NCBI short read archive using the fastq-dump function of the SRA-toolkit. In accordance with the published analysis, reads were trimmed to 90 bp, before quality control (discarding reads with a higher maximum expected error than 0.2 or positions with less than 13 quality score), error modelling (per project accession), ASV construction (per sample), table set-up, and taxonomic annotation (using the mothur [14] classifier). To handle the combined dataset table, 360 GB RAM were reserved for the final steps in R.
Efficiency was calculated as the ratio of CPU-time divided by the product of used slots and real walltime.

Databases

The SILVA [53] RefSSU_NR99 database v. 138 was used for the taxonomic classification of bacterial and archaean ASVs. Fungal ASVs were classified against the UNITE v8 database [57,58]. Both sets of ASVs were classified using the Bayesian classifier as implemented in mothur's classify.seqs command [14], with a cut-off of 60.

Visualization and statistics

The output of all dadasnake runs was gathered in an R-workspace (tabular version see Supplementary table 3). Rarefaction curves were plotted using vegan [33]. The coefficient of variation was calculated as the ratio of the standard deviation to the mean. The cluster-job information for the performance tests was gathered in an R-workspace. Efficiency was calculated as the ratio of CPU-time divided by the product of used slots and real walltime.

Availability of supporting source code and requirements

Project name: dadasnake
Project home page: https://github.com/a-h-b/dadasnake
Operating system(s): Linux
Programming language: Python, R, bash
Other requirements: anaconda or other conda package manager
License: GNU GPL-3.0
RRID:SCR_019149

Availability of supporting data

The raw sequencing data generated for this manuscript are accessible on NCBI's Sequence Read Archive under BioProject accession PRJNA626434. Processing results of the mock community data sets, the ground-truth mock community compositions, and the scripts to visualize the use case datasets are available from Zenodo [59]. The frozen version of dadasnake described in this manuscript is available from Zenodo [60].

List of abbreviations

OTU – operational taxonomic unit
ASV – amplicon sequence variants (=ESV)
ESV – exact sequence variants (=ASV)
rRNA – ribosomal RNA

Competing interests

The authors declare that they have no competing interests.

Funding

AH-B was funded by the German Centre for Integrative Biodiversity Research (iDiv) Halle-Jena-Leipzig of the German Research Foundation (DFG - FZT118, grant number, 202548816). CW acknowledges funding from the German Research Foundation (DFG - GFBio II, grant number BU 941/23-2).

Authors' contributions

Conceptualization, software, analysis, writing: AH-B; optimization and testing: CW; sequencing: BS. All authors contributed to the manuscript text and approve its contents.

Acknowledgements

The authors would like to acknowledge Kezia Goldmann and Julia Moll for testing early versions of the workflow; François Buscot for funding acquisition and providing resources; Guillaume Lentendu for discussions. Data processing has been performed at the High-Performance Computing (HPC) Cluster EVE, a joint effort of both the Helmholtz Centre for Environmental Research - UFZ and the German Centre for Integrative Biodiversity Research (iDiv) Halle-Jena-Leipzig and the authors thank Christian Krause and the other administrators for excellent support. Matthew Bakker is acknowledged for the generous provision of the fungal mock community.

References

1. Sogin ML, Morrison HG, Huber JA, Mark Welch D, Huse SM, Neal PR, et al. Microbial diversity in the deep sea and the underexplored "rare biosphere". Proc Natl Acad Sci USA. 2006;103:12115–20.

2. Callahan BJ, McMurdie PJ, Holmes SP. Exact sequence variants should replace operational taxonomic units in marker-gene data analysis. The ISME Journal. 2017;11:2639–43.

3. Glassman SI, Martiny JB. Ecological patterns are robust to use of exact sequence variants versus operational taxonomic units. 2018;:1–25.

4. Johnson JS, Spakowicz DJ, Hong B-Y, Petersen LM, Demkowicz P, Chen L, et al. Evaluation of 16S rRNA gene sequencing for species and strain-level microbiome analysis. Nature Communications. 2019;10:5029.

5. Brumfield KD, Huq A, Colwell RR, Olds JL, Leddy MB. Microbial resolution of whole genome shotgun and 16S amplicon metagenomic sequencing using publicly available NEON data. PLoS ONE. 2020;15:e0228899–21.

6. Hugerth LW, Andersson AF. Analysing Microbial Community Composition through Amplicon Sequencing: From Sampling to Hypothesis Testing. Front. Microbiol. 2017;8:23–22.

7. Nearing JT, Douglas GM, Comeau AM, Langille MGI. Denoising the Denoisers: an independent evaluation of microbiome sequence error-correction approaches. PeerJ. 2018;6:e5364–22.

8. Piwosz K, Shabarova T, Pernthaler J, Posch T, Simek K, Porcal P, et al. Bacterial and Eukaryotic Small-Subunit Amplicon Data Do Not Provide a Quantitative Picture of Microbial Communities, but They Are Reliable in the Context of Ecological Interpretations. mSphere. 2020;5:66–14.

9. Yeh Y-C, Needham DM, Sieradzki ET, Fuhrman JA. Taxon Disappearance from Microbiome Analysis Reinforces the Value of Mock Communities as a Standard in Every Sequencing Run. mSystems. 2018;3:337–9.

10. Tessler M, Neumann JS, Afshinnekoo E, Pineda M, Hersch R, Velho LFM, et al. Large-scale differences in microbial biodiversity discovery between 16S amplicon and shotgun sequencing. Scientific Reports. 2017;7:6589–14.

11. Rausch P, Rühlemann M, Hermes BM, Doms S, Dagan T, Dierking K, et al. Comparative analysis of amplicon and metagenomic sequencing methods reveals key features in the evolution of animal metaorganisms. Microbiome. 2019;7:133–19.

12. Thompson LR, Sanders JG, McDonald D, Amir A, Ladau J, Locey KJ, et al. A communal catalogue reveals Earth’s multiscale microbial diversity. Nature. 2017;551:457–63.

13. Bolyen E, Rideout JR, Dillon MR, Bokulich NA, Abnet CC, Al-Ghalith GA, et al. Reproducible, interactive, scalable and extensible microbiome data science using QIIME 2. Nat. Biotechnol. 2019;37:852–7.

14. Schloss PD, Westcott SL, Ryabin T, Hall JR, Hartmann M, Hollister EB, et al. Introducing mothur: open-source, platform-independent, community-supported software for describing and comparing microbial communities. Applied and Environmental Microbiology. 2009;75:7537–41.

15. Edgar RC. UPARSE: highly accurate OTU sequences from microbial amplicon reads. Nat Meth. 2013;10:996–8.

16. Rognes T, Flouri T, Nichols B, Quince C, Mahé F. VSEARCH: a versatile open source tool for metagenomics. PeerJ. 2016;4:e2584–22.

17. Hildebrand F, Tadeo R, Voigt AY, Bork P, Raes J. LotuS: an efficient and user-friendly OTU processing pipeline. Microbiome. 2014;2:30.

18. Mysara M, Njima M, Leys N, Raes J, Monsieurs P. From reads to operational taxonomic units: an ensemble processing pipeline for MiSeq amplicon sequencing data. GigaScience. 2017;6:1–10.

19. Zafeiropoulos H, Viet HQ, Vasileiadou K, Potirakis A, Arvanitidis C, Topalis P, et al. PEMA: a flexible Pipeline for Environmental DNA Metabarcoding Analysis of the 16S/18S ribosomal RNA, ITS, and COI marker genes. GigaScience. 2020;9:111–2.

20. Köster J, Rahmann S. Snakemake--a scalable bioinformatics workflow engine. Bioinformatics. 2012;28:2520–2.

21. Callahan BJ, McMurdie PJ, Rosen MJ, Han AW, Johnson AJA, Holmes SP. DADA2: High-resolution sample inference from Illumina amplicon data. Nat. Methods. 2016;13:581–3.

22. Pauvert C, Buée M, Laval V, Edel-Hermann V, Fauchery L, Gautier A, et al. Bioinformatics matters: The accuracy of plant and soil fungal community data is highly dependent on the metabarcoding pipeline. Fungal Ecology. 2019;41:23–33.

23. Caruso V, Song X, Asquith M, Karstens L. Performance of Microbiome Sequence Inference Methods in Environments with Varying Biomass. Gibbons SM, editor. mSystems. 2019;4:15–9.

24. Prodan A, Tremaroli V, Brolin H, Zwinderman AH, Nieuwdorp M, Levin E. Comparing bioinformatic pipelines for microbial 16S rRNA amplicon sequencing. Seo J-S, editor. PLoS ONE. 2020;15:e0227434–19.

25. Martin M. Cutadapt removes adapter sequences from high-throughput sequencing reads. EMBnet j. 2011;17:10.

26. Murali A, Bhargava A, Wright ES. IDTAXA: a novel approach for accurate taxonomic classification of microbiome sequences. Microbiome. 2018;6:140–14.

27. Wright ES. Using DECIPHER v2.0 to Analyze Big Biological Sequence Data in R. the R journal. 2016;8:352–9.

28. Camacho C, Coulouris G, Avagyan V, Ma N, Papadopoulos J, Bealer K, et al. BLAST+: architecture and applications. BMC Bioinformatics. 2009;10:421–9.

29. Bengtsson-Palme J, Ryberg M, Hartmann M, Branco S, Wang Z, Godhe A, et al. Improved software detection and extraction of ITS1 and ITS2 from ribosomal ITS sequences of fungi and other eukaryotes for analysis of environmental sequencing data. Methods in Ecology and Evolution. 2013;4:914–9.

30. Sievers F, Higgins DG. Clustal Omega, accurate alignment of very large numbers of sequences. Methods Mol. Biol. 2014;1079:105–16.

31. Price MN, Dehal PS, Arkin AP. FastTree 2--approximately maximum-likelihood trees for large alignments. PLoS ONE. 2010;5:e9490.

32. McMurdie PJ, Holmes S. phyloseq: an R package for reproducible interactive analysis and graphics of microbiome census data. PLoS ONE. 2013;8:e61217.

33. Oksanen J, Blanchet FG, Kindt R, Legendre P, Minchin PR, O'Hara RB, et al. vegan: Community Ecology Package. 2015.

34. Brown J, Zavoshy N, Brislawn CJ, McCue LA. Hundo: a Snakemake workflow for microbial community sequence data. PeerJ PrePrints. 2018;:e27272v1.

35. Boers SA, Hays JP, Jansen R. Micelle PCR reduces chimera formation in 16S rRNA profiling of complex microbial DNA mixtures. Scientific Reports. 2015;5:14181.

36. Callahan BJ, Wong J, Heiner C, Oh S, Theriot CM, Gulati AS, et al. High-throughput amplicon sequencing of the full-length 16S rRNA gene with single-nucleotide resolution. Nucleic Acids Res. 2019;13:360–12.

37. Li C, Chng KR, Boey EJH, Ng AHQ, Wilm A, Nagarajan N. INC-Seq: accurate single molecule reads using nanopore sequencing. GigaScience. 2016;5:34.

38. Calus ST, Ijaz UZ, Pinto AJ. NanoAmpli-Seq: a workflow for amplicon sequencing for mixed microbial communities on the nanopore sequencing platform. GigaScience. 2018;7:1–16.

39. BIOM community. The Biological Observation Matrix (BIOM) format. http://biom-format.org/index.html. Accessed 4 Nov 2020.

40. Heintz-Buschart A. dadasnake repository on github. http://github.coma-h-b/dadasnake. Accessed 4 Nov 2020.

41. Schleuss P-M, Widdig M, Heintz-Buschart A, Guhr A, Martin S, Kirkman K, et al. Stoichiometric controls of soil carbon and nitrogen cycling after long-term nitrogen and phosphorus addition in a mesic grassland in South Africa. Soil Biology and Biochemistry. 2019;135:294–303.

42. Leff JW, Jones SE, Prober SM, Barberán A, Borer ET, Firn JL, et al. Consistent responses of soil microbial communities to elevated nutrient inputs in grasslands across the globe. Proc Natl Acad Sci USA. 2015;112:10967–72.

43. D'Amore R, Ijaz UZ, Schirmer M, Kenny JG, Gregory R, Darby AC, et al. A comprehensive benchmarking study of protocols and sequencing platforms for 16S rRNA community profiling. BMC Genomics. 2016;17:55.

44. Bakker MG. A fungal mock community control for amplicon sequencing experiments. Mol Ecol Resour. 2018;18:541–56.

45. Salter SJ, Cox MJ, Turek EM, Calus ST, Cookson WO, Moffatt MF, et al. Reagent and laboratory contamination can critically impact sequence-based microbiome analyses. BMC Biol. 2014;12:87.

46. Thiéry O, Vasar M, Jairus T, Davison J, Roux C, Kivistik P-A, et al. Sequence variation in nuclear ribosomal small subunit, internal transcribed spacer and large subunit regions of *Rhizophagus irregularis* and *Gigaspora margarita* is high and isolate-dependent. Mol Ecol. 2016;25:2816–32.

47. Hurlbert SH. The Nonconcept of Species Diversity: A Critique and Alternative Parameters. Ecology. 1971;52:577–86.

48. O'Hara RB. Species richness estimators: how many species can dance on the head of a pin? J Anim Ecology. 2005;74:375–86.

49. Chiu C-H, Wang Y-T, Walther BA, Chao A. An improved nonparametric lower bound of species richness via a modified good-turing frequency formula. Biom. 2014;70:671–82.

50. Brach T. Callahan BJ. In dada2 default data (i.e. pooled = FALSE) richness correlates with total amplicons but rarefaction curves plateau early because the data usually contains almost no singletons. How to correctly correct for this effect? · Issue #317. Available from: https://github.com/benjjneb/dada2/issues/317. Accessed 4 Nov 2020.

51. Edgar RC. UNBIAS: An attempt to correct abundance bias in 16S sequencing, with limited success. bioRxiv. Cold Spring Harbor Laboratory; 2017;10:57–23.

52. D'Amore R, Ijaz UZ, Schirmer M, Kenny JG, Gregory R, Darby AC, et al. A comprehensive benchmarking study of protocols and sequencing platforms for 16S rRNA community profiling. BMC Genomics. BioMed Central; 2016;17:55.

53. Quast C, Pruesse E, Yilmaz P, Gerken J, Schweer T, Yarza P, et al. The SILVA ribosomal RNA gene database project: improved data processing and web-based tools. Nucleic Acids Research. 2013;41:D590–6.

54. White TJ, Bruns T, Lee S, Taylor JL. Amplification and direct sequencing of fungal ribosomal RNA genes for phylogenetics. PCR protocols: a guide to methods and applications. 1990;18:315–22.

55. Ihrmark K, Bödeker I, Cruz-Martinez K, Friberg H, Kubartova A, Schenck J, et al. New primers to amplify the fungal ITS2 region–evaluation by 454-sequencing of artificial and natural communities. FEMS Microbiol. Ecol. 2012;82:666–77.

56. Cruaud P, Rasplus J-Y, Rodriguez LJ, Cruaud A. High-throughput sequencing of multiple amplicons for barcoding and integrative taxonomy. Scientific Reports. 2017;:1–12.

57. Kõljalg U, Nilsson RH, Abarenkov K, Tedersoo L, Taylor AFS, Bahram M, et al. Towards a unified paradigm for sequence-based identification of fungi. Mol Ecol. 2013;22:5271–7.

58. Abarenkov, Kessy; Zirk, Allan; Piirmann, Timo; Pöhönen, Raivo; Ivanov, Filipp; Nilsson, R. Henrik; Kõljalg, Urmas (2020): UNITE general FASTA release for Fungi. Version 04.02.2020. UNITE Community. https://doi.org/10.15156/BIO/786368.

59. Heintz-Buschart A. Supplementary Datasets for dadasnake workflow [Dataset]. Zenodo; 2020. http://doi.org/10.5281/zenodo.4181260.

60. Heintz-Buschart A. a-h-b/dadasnake: Crictor (Version v0.7.2z) [Dataset]. Zenodo; 2020. http://doi.org/10.5281/zenodo.4190897.

Figure Legends

Figure 1: Overview of the dadasnake workflow for paired-end Illumina sequencing of a fungal ITS region with inputs (configuration file, sample table and read files) and outputs (read numbers, graphical representations of quality and error models, rarefaction curves and "OTU tables", in biom, table and phyloseq format). The steps are configurable and alternative workflows exist, e.g. for single-end, non-Illumina datasets, or other target regions. Primer-removal and all post-DADA2-steps are optional. Colours represent the level of analysis: yellow – analysis per library/sample, bright green – analysis per run, sea green – analysis of the cumulated dataset; blue – analysis for the whole dataset with sample-wise documentation; note – the DADA2 block can be performed in pooled mode at the level of the whole dataset.

Figure 2: Visualization of resource use by processing different datasets. a) The small (24 sample) 16S rRNA V4 amplicon dataset [41] processed linearly on a single core; b) the same dataset processed on up to four cores (each depicted as a vertical stack); c) a medium sized (267 sample) ITS1 amplicon dataset [42], processed on up to four cores; d) the same dataset, processed on up to 15 cores. Each block represents one job issued by dadasnake, colors represent the respective steps.

Figure 3: Comparison of mock community composition with analysis results. a) Detection of prokaryotic genera at the highest sequencing depth (1.6 mio reads); b) detection of fungal genera at the highest sequencing depth (40,000 reads); c) number of detected prokaryotic ASVs plotted against the number of processed (non-chimeric) reads - black circles: ASVs of taxa from the mock community, grey circles: likely contaminant taxa; d) number of detected fungal ASVs against the number of processed (non-chimeric) reads of the fungal mock community; c & d) dotted lines indicate expected taxa richness; e) missing correlation of real percentages of the mock communities and detected relative abundances of prokaryotic genera; f) coefficients of variation between relative abundances of taxa that should be equally abundant in the fungal mock community.

Supplementary Material

Supplementary file 1: Example of a YAML configuration file: configuration for the large dataset of the performance test.

Supplementary table 1: Description of all configurable settings.

Supplementary table 2: Description of outputs.

Supplementary table 3: Mock-community compositions and identification of ASVs from mock-community datasets.

Figure 1

Click here to access/download;Figu

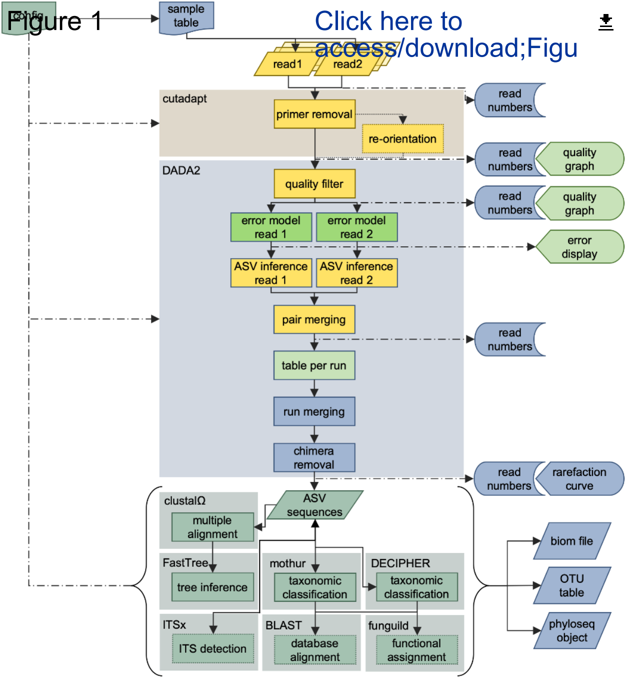

Figure 2

Click here to access/download;Figure;Figure2.pdf

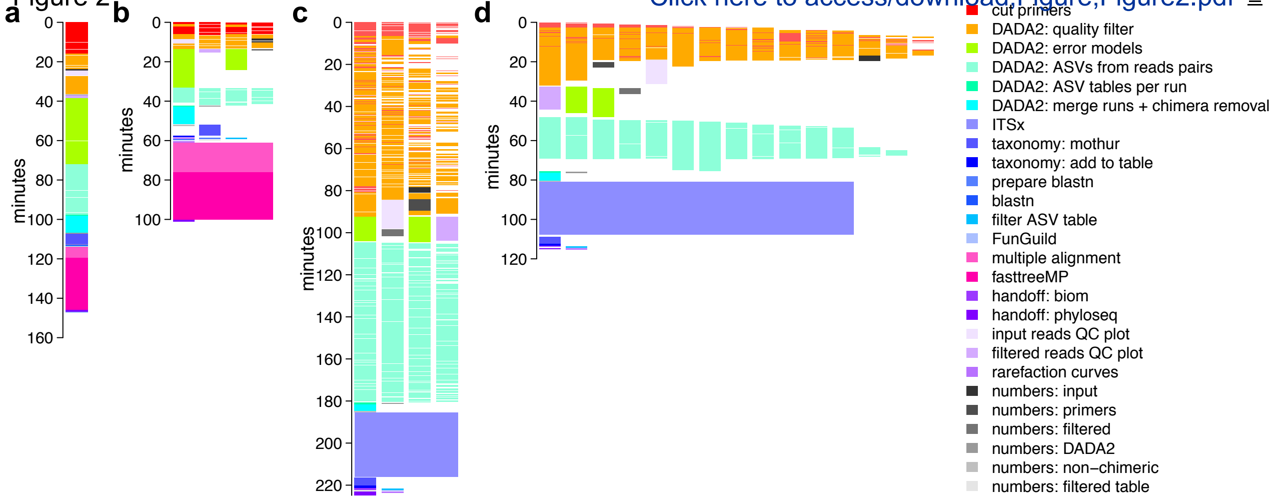

Figure 3

Click here to access/download;Figure;figure3.pdf

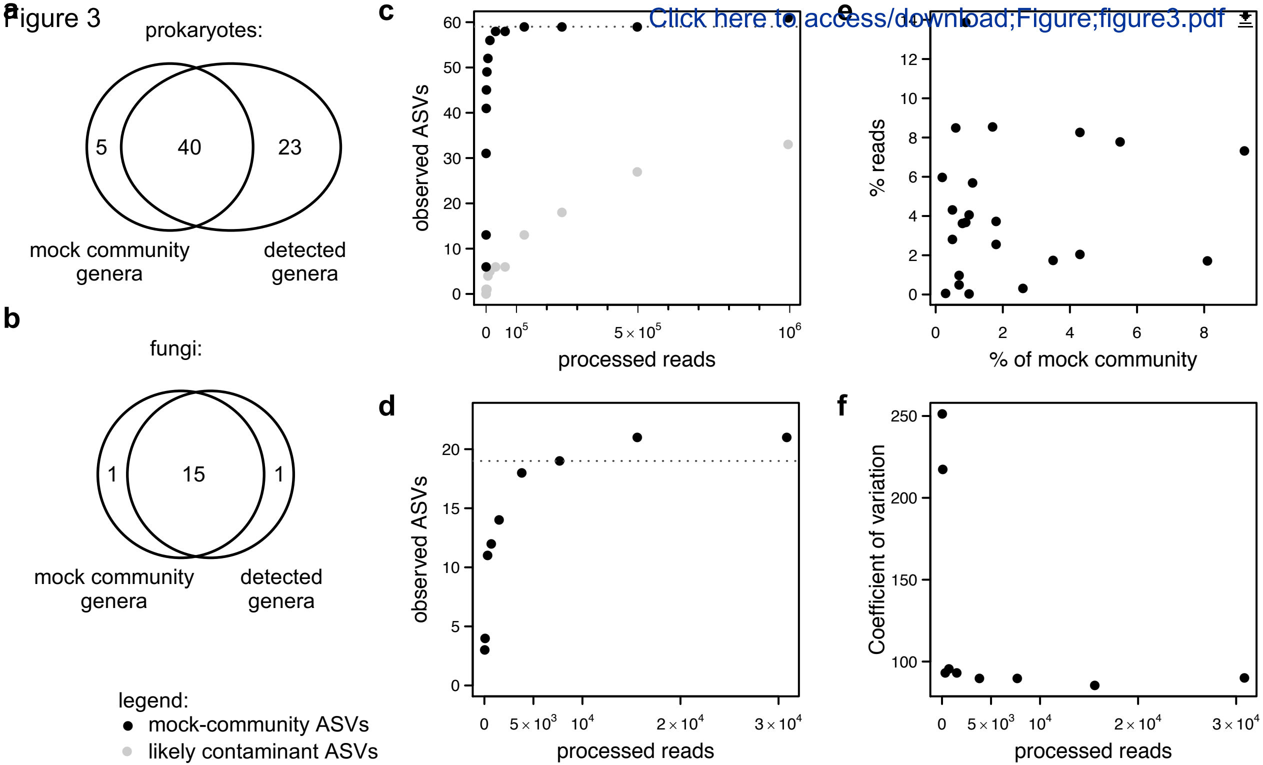

Supplementary File 1

Click here to access/download
Supplementary Material
LargeDataset.config.yaml

Supplementary Table 1

Click here to access/download
Supplementary Material
Supp_Table1_config_explained.xlsx

Supplementary Table 2

Click here to access/download
Supplementary Material
SupplementaryTable2_outputs.xlsx

Supplementary Table 3

Click here to access/download
Supplementary Material
supplementary_table3-groundTruthVsIdentification.xlsx

Click here to access/download
Supplementary Material
201015_draft.docx
