# Supplementary material for: Dadasnake, a Snakemake implementation of DADA2 to process amplicon sequencing data for microbial ecology
Source: Gigascience. 2020 Nov 30;9(12):giaa135. doi: 10.1093/gigascience/giaa135 (PMC7702218; doi:10.1093/gigascience/giaa135)
Supplement: giaa135_Response_to_Reviewer_Comments_Original_Submission [file giaa135_response_to_reviewer_comments_original_submission.pdf]

Author's Response To Reviewer Comments

Close

Dear Hongling Zhou, dear GigaScience Editors,

Please find enclosed our revision of manuscript GIGA-D-20-00147, now entitled “dadasnake, a Snakemake implementation of DADA2 to process amplicon sequencing data for microbial ecology”, which we would like to re-submit for your consideration and potential publication as Technical Note in GigaScience.

We have now addressed all comments by the two reviewers, in particular by adding better descriptions of how our “dadasnake” workflow can be used, by discussing how it is distinguished from other workflows, and by including several additional case studies to evaluate its performance and demonstrate that our “dadasnake” workflow provides a flexible, single-command pipeline to facilitate the efficient analysis of large-scale amplicon sequencing or metabarcoding data.

We have attached a point-by-point response to the reviewers’ valuable comments to this letter. In light of the reviewers’ overall positive response and the improvements made according to their statements, we are hopeful that you will agree on the revised manuscripts’ interest for the readers of GigaScience and find it suitable for publication.

We have also submitted and revised the manuscript as a preprint on bioRxiv (BIORXIV/2020/095679).

Thank you in again for considering our manuscript, and we look forward to hearing from you.

Sincerely,

Anna Heintz-Buschart

Editor's and reviewers' comments below:

Your manuscript "Efficiently processing amplicon sequencing data for microbial ecology with dadasnake, DADA2 in Snakemake" (GIGA-D-20-00147) has been assessed by our reviewers. Both reviewers have requested a lot of additional work. In particular this work needs a better explanation and description on how to use it. As well as additional testing and comparisons to meet our criteria on computational work meeting state-of-the-art. We are giving you a chance to address these points, but if you need additional time we can close your file and await a resubmission later.

Reviewer reports:

Reviewer #1: General comments: In the manuscript, the authors presented a wrapper, dadasnake, for existing softwares to analyze amplicon data. I appreciate that dadasnake can facilitate the community analysis of the amplicon datasets. Also, I appreciate that the workflow was tested on a fungal mock community sequenced for this paper. However, I would like to address some concerns, mainly about the novelty of the study and benchmarking results. Other pipelines, both in snakemake (e.g. https://github.com/shu251/tagseq-qiime2-snakemake) or other platforms (i.e. https://doi.org/10.7287/peerj.preprints.27272v1), are available for the processing of amplicon datasets with dada2. Therefore, I would ask the authors to specify more about the novel aspect that dadasnake brings to the microbiome community, and to compare to existing pipelines. Regarding this, you may find my assessments on a number of aspects recommended by GigaScience reviewer guidelines. Also, at the end of the text I provided some minor specific comments.

Answer: We thank you for your appreciation of the dadasnake workflow and the recommendations for improving the manuscript. We have addressed all points in the manuscript as well as below, especially as regards the differences to the many other workflows for amplicon sequencing data analysis.

We would like to particularly thank you for pointing us to other amplicon sequencing analysis pipelines with in snakemake, which we would have liked to reference in the manuscript. While we don't wish to discuss the details in the manuscript, we'd like to point out that we could not run the tagseq-qiime2-snake pipeline due to undocumented dependencies.

1. Is the rationale for collecting and analyzing the data well defined?

The work describes fairly well the previous work on the field. I appreciate that the authors sequence a house-built fungal community for testing their workflow.

Answer: We are very sorry, but we have not built the fungal community ourselves. The source of the fungal mock-community was clearly stated in the manuscript, and we have pointed to this again in the methods section to avoid the impression that we did build the community (L 232).

2. Is it clear how data was collected and curated?

Some details about composing the fungal mock community are missing (e.g. Which fungi are there, what are the percentage abundances of each fungi and how did you count and merge fungal cells or DNA?).

Answer: As explained above, the mock community was obtained from Matt Bakker, who has detailed this in the cited publication (ref 42 in revised reference counting). We had previously mentioned the fact that it is even, i.e. the same amount of each fungal species should be present, in the results section (L 203) and now mention it additionally in the methods section (L 232). The list of species in both analysed mock-communities had already been included in the supplementary materials, and we now point to this in the results section (L 162, 178) and methods section (L 217, 233).

Descriptions of some analysis are also missing (e.g. coefficient of variation). Otherwise, the workflow is explained well.

Answer: Thank you, also for pointing out this omission, we've added the equation in L 299-300.

3. Is it clear - and was a statement provided - on how data and analyses tools used in the study can be accessed?
All the scripts are available in the github page of dadasnake. Also, accession numbers for the data are provided.

Answer: Thank you.

4. Are accession numbers given or links provided for data that, as a standard, should be submitted to a community approved public repository?

Accession numbers are provided for the data.

Answer: Thank you.

5. Is the data and software available in the public domain under a Creative Commons license?

The code is stored in a github repository with a GNU general public license.

Answer: Thank you.

6. Are the data sound and well controlled?

The details about the archaea part of the mock community is missing.

Answer: We are very sorry for lumping archaea and bacteria together – we do know better. We have now reported them separately (L 161, 164, 217).

7. Is the interpretation (Analysis and Discussion) well balanced and supported by the data?

Overall, current literature, results and limitations of the study interpreted and discussed well. The

authors also provided references wherever necessary.

Answer: Thank you for this appraisal.

However, benchmarking the accuracy of the wrapper might be unnecessary (Figure2-3) since the wrapper relies on softwares and packages whose accuracy is already tested (e.g. dada2, ITSx). In my opinion, the main point of the wrapper here is not to increase the accuracy but to facilitate the usage of the amplicon analysis pipelines. Therefore, it would be more interesting to compare dadasnake to vanilla dada2 (or other dada2 implementations e.g. in QIIME2), and to compare dadasnake to other OTU pipelines such as QIIME2, mothur, LotuS etc.

Answer: Thank you for this suggestion. We agree that the accuracy of DADA2 has already been evaluated several times, which is why we're using it in dadasnake – this is also the same whether wrapped in QIIME2 or in vanilla R. We feel that it is nevertheless important to demonstrate that the pipeline as a whole produces realistic results and provide ideas on the limitations of the process in the manuscript.
Regarding ease of use, this is difficult to assess objectively and depends strongly on the background of the users. However, according to your comment, we have now included a discussion of the points where dadasnake is easier to use than other pipelines and where it is more flexible (L 63-65, 94-120).

One main component is missing: the computational time, memory usage, hdd usage is not discussed at all and could be used instead of the current Fig. 3 to illustrate the efficiency of the pipeline.

Answer: We have now added examples for three differently sized datasets and report the resource use in a new paragraph and figure (L 125-156, Figure 2).

8. Are the methods appropriate, well described, and include sufficient details and supporting information to allow others to evaluate and replicate the work?

Methods for the workflow are well described. Also, in the github page, parameters are well-explained and possible user errors are stated. However, some points in the methods and results sections deserve more attention which we explained at 7 and 9.

Answer: Thank you. Please see our responses in 1,2,6,7,9 for those points.

9. What are the strengths and weaknesses of the methods?
Bacterial and archaean mock community data set:
What happened to the archeal reads? I did not see that it is reported further in the results, this should be further discussed.

Answer: We have revised this, as mentioned in point 6 above.

Fungal mock community sequencing:
Was ITSx also used in the analysis? It was mentioned in the implementation section; however, it was not mentioned in the analysis of the fungal mock community and how it performed/helped. Also, could you please provide further details on composing of the mock community as we mentioned at point 2?

Answer: Indeed, we used ITSx in the analysis of the fungal mock community. In the case of the mock community, it doesn't have an effect on the final result. It is however useful when the real dataset contains non-fungal ASVs, which is often the case in real samples, e.g. from soil. We've now added this detail (L 248).

10. Have the authors followed best-practices in reporting standards?
The methods used in the dadasnake workflow are amenable. Also, the authors provided the conda environment for dadasnake, which facilitates easy installation.

Answer: Thank you.

11. Can the writing, organization, tables and figures be improved?

The quality of the written English is amenable and the manuscript is organized in a logical way. However, figures may be made more comprehensive:

Figure2: Why ASVs from the fungal mock community are not denoted in Figure 2d?

Answer: Figure 3d (formerly figure 2d) does show the numbers of fungal ASVs detected and expected from the fungal mock community. The legend of this figure has been clarified now.

Also, bacterial false positives are too many, the results in the dada2 paper looked a lot better and this might be a very interesting discussion to add to the paper. How to be sure that this is mainly due to contamination? Please consider rephrasing "likely contaminants" - what is the evidence for this? Please discuss this in the manuscript.

Answer: We were also originally surprised by the number of false positives, especially compared to earlier benchmarks. We've checked the distance between the potential contaminant ASVs and the nearest ASVs from expected taxa and found them to be too unrelated to be down to sequencing errors. We are confident that these are not artificial ASVs but represent contaminating DNA. As we've previously stated, this has been observed before. We now discuss in some more detail (L 165-169) and have added a column in the results in supplementary table 3 to indicate previously established contaminants.
Furthermore, the ASVs are classified to genus or species level, which we think would be highly unlikely if they were artificial ASVs – we don't have a reference or analysis to back this specific claim up, so we are not discussing the exact classification in the manuscript.

Overall, Figure 2 could be clearer, please consider adding a legend etc.

Answer: Thank you for the comment. We've reworked this figure when we merged it with figure 3 (now figure 3).

Figure 3: I am not sure whether Figure3 A-B adds further value to the manuscript, considering Figure 2C-D.

Answer: We've combined Figure 3c/d with Figure 2 (now Figure 3) and removed Figure 3a/b. Instead, we point to the DADA2 documentation, which mentions this effect, too (L 193).

Figure 3c) Please explain better what is x- axis: "part" of mock community.

Answer: Thank you for pointing out that this is unclear, we've changed the axis label to read "percentage of mock-community" now (Fig 3 e).

Figure 3D) How did you calculate a coefficient of variation? Why not make Figure 3C in the same way?

Answer: The reason to use the coefficient of variation is that we expect equal amounts for the fungal mock community. Compared to Fig. 3E, we'd have the same value along the x-axis for all taxa. We've made this more clear in the text now (L 201-204).

12. When revisions are requested.

Minor revision is requested.

Answer: Thank you.

13. Are there any ethical or competing interests issues you would like to raise?

Not detected.

Specific Comments:
Full title: Why efficient? The efficiency of the pipeline is not really tested in this study. This should be benchmarked against other pipelines (even those not using dada2 as clustering), and against vanilla dada2.

Answer: Thank you for this suggestion. We've adjusted the title accordingly.

Abstract: line2-3: what do you mean by "structure of microbial communities". Please use a less ambiguous term.

Answer: Thank you for pointing out that this term that it well established in microbial ecology is not informative to most readers of GigaScience. We've translated it to standard English now.

Background:
L7: lack of quantitative value → lack of absolute abundances

Answer: Thank you for this suggestion. We've adjusted the sentence accordingly (L 45).

L8: an overview of microbial diversity and composition

Answer: Thank you for this suggestion. We've adjusted the sentence accordingly (L 46).

"A second limitation is that relative abundances of ASVs are not reflective of the actual abundance of the sequenced taxa, which varied for the prokaryotic mock community and were equal in the fungal mock community." → this is rather a limitation of the amplicon sequencing.

Answer: Thank you for this suggestion. We've adjusted the sentence accordingly (L 199).

Reviewer #2: "Dadasnake" is a snakemake workflow that implements the DADA2 method for processing amplicon sequencing data, as well as additional tools for assigning taxonomy, creating phylogenetic trees, and inferring function from denoised tables of common taxonomic barcode genes (e.g. 16S, ITS). The benefits of the dadasnake workflow are in its combination of snakemake, which efficiently and reproducibly makes use of high-performance computing (HPC) resources, with widely used and well-validated amplicon sequencing tools that would otherwise be difficult for many to easily and effectively use in an HPC environment. The goal of this project is laudable. The documentation around some of the core tools incorporated here, in particular DADA2, are mostly centered around operation on a personal computer, and for non-experts the deployment of these tools on HPCs can be a bridge too far.

Answer: Thank you for your acknowledgement of dadasnake's purpose.

However, I think the current manuscript suffers from a critical weakness in that the dadasnake workflow itself is not sufficiently explained and explored, particularly when it comes to the ways in which potential (non-expert) users might interact with it while configuring parameters, assessing whether processing happened as expected, and diagnosing problems when they arise. There is significantly more information on these critical topics in the online documentation available at https://github.com/a-h-b/dadasnake, which is fine for the details. But… I think the paper itself needs a clear description of parameterization including the yaml file format that would allow a non-expert to understand how they might use that format to change relevant parameters, a fuller description of the intermediate outputs/visualizations that snakemake or tools like DADA2 create within the dada2snake workflow that would allow a non-expert to understand how they might go about fixing poorly chosen parameters or diagnosing the step at which the workflow failed, and what they could do once something has gone wrong.

Answer: Thank you for this suggestion. We've originally wanted to keep the manuscript short. We now realize that we have to bridge between the short description and the documentation in the github repository. We have now expanded the section on configuring and running dadasnake (L 95-101, 111-123), including more explicit references to the supplementary material and additional explanatory supplementary tables. More detailed information will still best be accessed via the github page, which has the added advantage of us being able to update the information according to users' comments.

Furthermore, some evaluation of the ability of dadasnake to efficiently use HPC hardware would be another very nice element. Simple question: How much does wall-time decrease when using dadasnake on 4 nodes vs. 40 nodes? More complex question: Are there particular steps that become rate limiting in very high resource environments (maybe tree-ing?).

Answer: Thank you for this suggestion. We have added information on the resource use of dadasnake (L

125-155, 250-289).

The paragraph above is my biggest concern. Other itemized issues/suggestions below, with *(s) appending those that are more important.

** What is the plan for the maintenance or continued development of the conda environment behind dadasnake? Is it currently pinned to defined versions of each package? Or will it update along with the packages as they appear on bioconda? How will testing of package updates be done?

Answer: Thank you for this suggestion. The workflow is indeed pinned to fixed versions of each package. Since we are heavily using the workflow, it's in our own interest to update and test when new versions, in particular of DADA2 become available. For example, the latest version of dadasnake has moved to R4.0.2. We have added our future plans in L 87-89.

* What is the user support plan when folks have problems with the workflow? The documentation at the website is acceptable, but I guarantee there will be problems folks have they won't be able to solve via the documentation. What then?

Answer: Since the original submission, we have expanded the documentation in the github page, including trouble-shooting. We've also attended to issues posted there.

* Default parameter choices become the parameters used by the vast majority of any workflow. How were the defaults for dadasnake chosen? I can see at least one default parameter (truncQ=13) that is not the default suggested by the tool itself.

Answer: Thank you for pointing out this difference. According to your comment, we've changed the DADA2 default settings within dadasnake to the DADA2 defaults (in particular in the filtering step, where they differed from the DADA2 defaults).
We've chosen the settings in the example config files based on the accuracy of the results in the reported (and other) mock datasets. We've kept these changed parameters in the example configurations, as they work best in our hands.

"Pooled analysis can alternatively be chosen in dadasnake and is recommended for more error prone technologies such as 454 or third generation long reads.": Is this the authors recommendation? If so say "we recommend". If not, can the authors provide a source or citation for this recommendation?

Answer: We've edited the sentence according to your suggestion (L 104-106). It is our observation that non-pooled analysis of error-prone reads leads to different ASVs in each sample. We actually provide a mock community dataset from 454 sequencing in the supplementary material (now referenced in L 108). However, we don't want to inflate the manuscript with information that will not be of interest to most users, so we've not discussed it further.

"Exact sequencing variants" -> "exact sequence variants", several places.

Answer: Oh dear, we're sorry and have corrected this throughout.

Snakemake is central to this whole thing. Perhaps it should be a keyword, and perhaps should be described a bit more in the "Implementation" section.

Answer: We've expanded the Snakemake description in the usage paragraph (L 84-91, 118-121). Snakemake is part of the title, so it'll be visible for searches and bibliographical applications.

Use case/Limitations: As I mentioned above, this is narrowly focused on how DADA2/amplicon sequencing did at measuring a mock community, and this tells us little about how dadasnake performs, other than it can successfully run DADA2 on a single test dataset.

Answer: According to your and the other reviewer's comment, we've added useful information on the different scales of datasets dadasnake can handle (L 125-156, 250-289).

Close
