# Supplementary material for: Dadasnake, a Snakemake implementation of DADA2 to process amplicon sequencing data for microbial ecology
Source: Gigascience. 2020 Nov 30;9(12):giaa135. doi: 10.1093/gigascience/giaa135 (PMC7702218; doi:10.1093/gigascience/giaa135)
Supplement: giaa135_Reviewer_1_Report_Original_Submission — Falk Hildebrand -- 6/13/2020 Reviewed [file giaa135_reviewer_1_report_original_submission.pdf]

Reviewer Report

Title: dadasnake, a Snakemake implementation of DADA2 to process amplicon sequencing data for microbial ecology

Version: Original Submission **Date:** 6/13/2020

Reviewer name: Falk Hildebrand

Reviewer Comments to Author:

General comments: In the manuscript, the authors presented a wrapper, dadasnake, for existing softwares to analyze amplicon data. I appreciate that dadasnake can facilitate the community analysis of the amplicon datasets. Also, I appreciate that the workflow was tested on a fungal mock community sequenced for this paper. However, I would like to address some concerns, mainly about the novelty of the study and benchmarking results. Other pipelines, both in snakemake (e.g. https://github.com/shu251/tagseq-qiime2-snakemake) or other platforms (i.e. https://doi.org/10.7287/peerj.preprints.27272v1), are available for the processing of amplicon datasets with dada2. Therefore, I would ask the authors to specify more about the novel aspect that dadasnake brings to the microbiome community, and to compare to existing pipelines. Regarding this, you may find my assessments on a number of aspects recommended by GigaScience reviewer guidelines. Also, at the end of the text I provided some minor specific comments.

1. Is the rationale for collecting and analyzing the data well defined?

The work describes fairly well the previous work on the field. I appreciate that the authors sequence a house-built fungal community for testing their workflow.

2. Is it clear how data was collected and curated?

Some details about composing the fungal mock community are missing (e.g. Which fungi are there, what are the percentage abundances of each fungi and how did you count and merge fungal cells or DNA?). Descriptions of some analysis are also missing (e.g. coefficient of variation). Otherwise, the workflow is explained well.

3. Is it clear - and was a statement provided - on how data and analyses tools used in the study can be accessed?

All the scripts are available in the github page of dadasnake. Also, accession numbers for the data are provided.

4. Are accession numbers given or links provided for data that, as a standard, should be submitted to a community approved public repository?

Accession numbers are provided for the data.

5. Is the data and software available in the public domain under a Creative Commons license?

The code is stored in a github repository with a GNU general public license.

6. Are the data sound and well controlled?

The details about the archaea part of the mock community is missing.

7. Is the interpretation (Analysis and Discussion) well balanced and supported by the data?

Overall, current literature, results and limitations of the study interpreted and discussed well. The

authors also provided references wherever necessary.
However, benchmarking the accuracy of the wrapper might be unnecessary (Figure2-3) since the wrapper relies on softwares and packages whose accuracy is already tested (e.g. dada2, ITSx). In my opinion, the main point of the wrapper here is not to increase the accuracy but to facilitate the usage of the amplicon analysis pipelines. Therefore, it would be more interesting to compare dadasnake to vanilla dada2 (or other dada2 implementations e.g. in QIIME2), and to compare dadasnake to other OTU pipelines such as QIIME2, mothur, LotuS etc.
One main component is missing: the computational time, memory usage, hdd usage is not discussed at all and could be used instead of the current Fig. 3 to illustrate the efficiency of the pipeline.
8. Are the methods appropriate, well described, and include sufficient details and supporting information to allow others to evaluate and replicate the work?
Methods for the workflow are well described. Also, in the github page, parameters are well-explained and possible user errors are stated. However, some points in the methods and results sections deserve more attention which we explained at 7 and 9.
9. What are the strengths and weaknesses of the methods?
Bacterial and archaean mock community data set:
What happened to the archeal reads? I did not see that it is reported further in the results, this should be further discussed.
Fungal mock community sequencing:
Was ITSx also used in the analysis? It was mentioned in the implementation section; however, it was not mentioned in the analysis of the fungal mock community and how it performed/helped. Also, could you please provide further details on composing of the mock community as we mentioned at point 2?
10. Have the authors followed best-practices in reporting standards?
The methods used in the dadasnake workflow are amenable. Also, the authors provided the conda environment for dadasnake, which facilitates easy installation.
11. Can the writing, organization, tables and figures be improved?
The quality of the written English is amenable and the manuscript is organized in a logical way. However, figures may be made more comprehensive:
Figure2: Why ASVs from the fungal mock community are not denoted in Figure 2d? Also, bacterial false positives are too many, the results in the dada2 paper looked a lot better and this might be a very interesting discussion to add to the paper. How to be sure that this is mainly due to contamination? Please consider rephrasing "likely contaminants" - what is the evidence for this? Please discuss this in the manuscript. Overall, Figure 2 could be clearer, please consider adding a legend etc.
Figure 3: I am not sure whether Figure3 A-B adds further value to the manuscript, considering Figure 2C-D.
Figure 3c) Please explain better what is x- axis: "part" of mock community.
Figure 3D) How did you calculate a coefficient of variation? Why not make Figure 3C in the same way?
12. When revisions are requested.
Minor revision is requested.
13. Are there any ethical or competing interests issues you would like to raise?
Not detected.
Specific Comments:

Full title: Why efficient? The efficiency of the pipeline is not really tested in this study. This should be benchmarked against other pipelines (even those not using dada2 as clustering), and against vanilla dada2.
Abstract: line2-3: what do you mean by "structure of microbial communities". Please use a less ambiguous term.
Background:
L7: lack of quantitative value â†’ lack of absolute abundances
L8: an overview of microbial diversity and composition
"A second limitation is that relative abundances of ASVs are not reflective of the actual abundance of the sequenced taxa, which varied for the prokaryotic mock community and were equal in the fungal mock community." â†’ this is rather a limitation of the amplicon sequencing.

Level of Interest

Please indicate how interesting you found the manuscript: Choose an item.

Quality of Written English

Please indicate the quality of language in the manuscript: Choose an item.

Declaration of Competing Interests

Please complete a declaration of competing interests, considering the following questions:

- Have you in the past five years received reimbursements, fees, funding, or salary from an organisation that may in any way gain or lose financially from the publication of this manuscript, either now or in the future?
- Do you hold any stocks or shares in an organisation that may in any way gain or lose financially from the publication of this manuscript, either now or in the future?
- Do you hold or are you currently applying for any patents relating to the content of the manuscript?
- Have you received reimbursements, fees, funding, or salary from an organization that holds or has applied for patents relating to the content of the manuscript?
- Do you have any other financial competing interests?
- Do you have any non-financial competing interests in relation to this paper?

If you can answer no to all of the above, write 'I declare that I have no competing interests' below. If your reply is yes to any, please give details below.

I declare that I have no competing interests

I agree to the open peer review policy of the journal. I understand that my name will be included on my report to the authors and, if the manuscript is accepted for publication, my named report including any attachments I upload will be posted on the website along with the authors' responses. I agree for my report to be made available under an Open Access Creative Commons CC-BY license (http://creativecommons.org/licenses/by/4.0/). I understand that any comments which I do not wish to be included in my named report can be included as confidential comments to the editors, which will not be published.

Choose an item.

To further support our reviewers, we have joined with Publons, where you can gain additional credit to further highlight your hard work (see: https://publons.com/journal/530/gigascience). On publication of this paper, your review will be automatically added to Publons, you can then choose whether or not to claim your Publons credit. I understand this statement.

Yes Choose an item.
