# Supplementary material for: Dadasnake, a Snakemake implementation of DADA2 to process amplicon sequencing data for microbial ecology
Source: Gigascience. 2020 Nov 30;9(12):giaa135. doi: 10.1093/gigascience/giaa135 (PMC7702218; doi:10.1093/gigascience/giaa135)
Supplement: giaa135_Reviewer_2_Report_Original_Submission — Benjamin Callahan -- 6/19/2020 Reviewed [file giaa135_reviewer_2_report_original_submission.pdf]

Reviewer Report

Title: dadasnake, a Snakemake implementation of DADA2 to process amplicon sequencing data for microbial ecology

Version: Original Submission **Date:** 6/19/2020

Reviewer name: Benjamin Callahan

Reviewer Comments to Author:

"Dadasnake" is a snakemake workflow that implements the DADA2 method for processing amplicon sequencing data, as well as additional tools for assigning taxonomy, creating phylogenetic trees, and inferring function from denoised tables of common taxonomic barcode genes (e.g. 16S, ITS). The benefits of the dadasnake workflow are in its combination of snakemake, which efficiently and reproducibly makes use of high-performance computing (HPC) resources, with widely used and well-validated amplicon sequencing tools that would otherwise be difficult for many to easily and effectively use in an HPC environment. The goal of this project is laudable. The documentation around some of the core tools incorporated here, in particular DADA2, are mostly centered around operation on a personal computer, and for non-experts the deployment of these tools on HPCs can be a bridge too far.
However, I think the current manuscript suffers from a critical weakness in that the dadasnake workflow itself is not sufficiently explained and explored, particularly when it comes to the ways in which potential (non-expert) users might interact with it while configuring parameters, assessing whether processing happened as expected, and diagnosing problems when they arise. There is significantly more information on these critical topics in the online documentation available at https://github.com/a-h-b/dadasnake, which is fine for the details. But… I think the paper itself needs a clear description of parameterization including the yaml file format that would allow a non-expert to understand how they might use that format to change relevant parameters, a fuller description of the intermediate outputs/visualizations that snakemake or tools like DADA2 create within the dada2snake workflow that would allow a non-expert to understand how they might go about fixing poorly chosen parameters or diagnosing the step at which the workflow failed, and what they could do once something has gone wrong. Furthermore, some evaluation of the ability of dadasnake to efficiently use HPC hardware would be another very nice element. Simple question: How much does wall-time decrease when using dadasnake on 4 nodes vs. 40 nodes? More complex question: Are there particular steps that become rate limiting in very high resource environments (maybe tree-ing?).
The paragraph above is my biggest concern. Other itemized issues/suggestions below, with *(s) appending those that are more important.
** What is the plan for the maintenance or continued development of the conda environment behind dadasnake? Is it currently pinned to defined versions of each package? Or will it update along with the packages as they appear on bioconda? How will testing of package updates be done?
* What is the user support plan when folks have problems with the workflow? The documentation at the website is acceptable, but I guarantee there will be problems folks have they won't be able to solve via the documentation. What then?

* Default parameter choices become the parameters used by the vast majority of any workflow. How were the defaults for dadasnake chosen? I can see at least one default parameter (truncQ=13) that is not the default suggested by the tool itself.
"Pooled analysis can alternatively be chosen in dadasnake and is recommended for more error prone technologies such as 454 or third generation long reads.": Is this the authors recommendation? If so say "we recommend". If not, can the authors provide a source or citation for this recommendation?
"Exact sequencing variants" -> "exact sequence variants", several places.
Snakemake is central to this whole thing. Perhaps it should be a keyword, and perhaps should be described a bit more in the "Implementation" section.
Use case/Limitations: As I mentioned above, this is narrowly focused on how DADA2/amplicon sequencing did at measuring a mock community, and this tells us little about how dadasnake performs, other than it can successfully run DADA2 on a single test dataset.

Level of Interest

Please indicate how interesting you found the manuscript: Choose an item.

Quality of Written English

Please indicate the quality of language in the manuscript: Choose an item.

Declaration of Competing Interests

Please complete a declaration of competing interests, considering the following questions:

- Have you in the past five years received reimbursements, fees, funding, or salary from an organisation that may in any way gain or lose financially from the publication of this manuscript, either now or in the future?
- Do you hold any stocks or shares in an organisation that may in any way gain or lose financially from the publication of this manuscript, either now or in the future?
- Do you hold or are you currently applying for any patents relating to the content of the manuscript?
- Have you received reimbursements, fees, funding, or salary from an organization that holds or has applied for patents relating to the content of the manuscript?
- Do you have any other financial competing interests?
- Do you have any non-financial competing interests in relation to this paper?

If you can answer no to all of the above, write 'I declare that I have no competing interests' below. If your reply is yes to any, please give details below.

I declare that I have no competing interests

I agree to the open peer review policy of the journal. I understand that my name will be included on my report to the authors and, if the manuscript is accepted for publication, my named report including any attachments I upload will be posted on the website along with the authors' responses. I agree for my report to be made available under an Open Access Creative Commons CC-BY license (http://creativecommons.org/licenses/by/4.0/). I understand that any comments which I do not wish to be included in my named report can be included as confidential comments to the editors, which will not be published.

Choose an item.

To further support our reviewers, we have joined with Publons, where you can gain additional credit to further highlight your hard work (see: https://publons.com/journal/530/gigascience). On publication of this paper, your review will be automatically added to Publons, you can then choose whether or not to claim your Publons credit. I understand this statement.

Yes Choose an item.
